# The membrane-associated form of cyclin D1 enhances cellular invasion

**DOI:** 10.1038/s41389-020-00266-y

**Published:** 2020-09-18

**Authors:** Ke Chen, Xuanmao Jiao, Anthony Ashton, Agnese Di Rocco, Timothy G. Pestell, Yunguang Sun, Jun Zhao, Mathew C. Casimiro, Zhiping Li, Michael P. Lisanti, Peter A. McCue, Duanwen Shen, Samuel Achilefu, Hallgeir Rui, Richard G. Pestell

**Affiliations:** 1 Department of Cancer Biology, Thomas Jefferson University, Philadelphia, PA 19107 USA; 2 Pennsylvania Cancer and Regenerative Medicine Research Center, Baruch S. Blumberg Institute, Pennsylvania Biotechnology Center, Wynnewood, PA 19096 USA; 3 Department of Pathology, Medical College of Wisconsin, Milwaukee, WI 53226 USA; 4 Dept of Science and Math, Abraham Baldwin Agricultural college, Tifton, GA 31794 Georgia; 5 Biomedical Research Centre (BRC), Translational Medicine, School of Environment and Life Sciences, University of Salford, Manchester, United Kingdom; 6 Department of Pathology, Anatomy and Cell Biology, Sidney Kimmel Cancer Center, Thomas Jefferson University, Philadelphia, PA 19107 USA; 7 Departments of Biomedical Engineering, Washington University, St. Louis, MO 63110 USA; 8 Departments of Radiology, Washington University, St. Louis, MO 63110 USA; 9 Departments of Biochemistry & Molecular Biophysics, Washington University, St. Louis, MO 63110 USA; 10 The Wistar Cancer Center, Wistar Institute, Philadelphia, PA 19104 USA

**Keywords:** Breast cancer, Cell signalling, Cell migration

## Abstract

The essential G_1_-cyclin, *CCND1*, is a collaborative nuclear oncogene that is frequently overexpressed in cancer. D-type cyclins bind and activate CDK4 and CDK6 thereby contributing to G_1_–S cell-cycle progression. In addition to the nucleus, herein cyclin D1 was also located in the cytoplasmic membrane. In contrast with the nuclear-localized form of cyclin D1 (cyclin D1^NL^), the cytoplasmic membrane-localized form of cyclin D1 (cyclin D1^MEM^) induced transwell migration and the velocity of cellular migration. The cyclin D1^MEM^ was sufficient to induce G_1_–S cell-cycle progression, cellular proliferation, and colony formation. The cyclin D1^MEM^ was sufficient to induce phosphorylation of the serine threonine kinase Akt (Ser473) and augmented extranuclear localized 17β-estradiol dendrimer conjugate (EDC)-mediated phosphorylation of Akt (Ser473). These studies suggest distinct subcellular compartments of cell cycle proteins may convey distinct functions.

## Introduction

The cyclin D1 (*CCND1*) gene, encodes the regulatory subunit of a holoenzyme that phosphorylates and inactivates the retinoblastoma protein (pRB), in order to promote cell cycle progression^1–3^. Newly synthesized cyclin D1 associates with CDK4/6 to form the holoenzyme that phosphorylates pRB, releasing E2F family transcription factors and inducing a gene expression network contributing to G_1_/S entry. Early studies demonstrated that cyclin D1 functions as a nuclear collaborative oncogene^4^. In this regard a cyclin D1 cDNA clone contributed to cellular transformation by complementing a transformation defective adenovirus E1A oncogene^4^. The requirement for cyclin D1 in oncogenic transformation has been established through cyclin D1 anti-sense^5,6^ and genetic deletion studies in the mouse^7–9^. Furthermore, cyclin D1 targeted to the mammary gland was sufficient for the induction of mammary tumorigenesis^10,11^. Clinical studies have shown a correlation between cyclin D1 expression and tumorigenesis and increased cyclin D1 expression is associated with tumor invasion and metastasis^12–15^.

A growing body of evidence provides support for an extranuclear function of cyclin D1. Cyclin D1 is actively synthesized and located exclusively in an extranuclear location in hibernating hematopoietic stem cells (HSC)^16^, in postmitotic neurons^17^, cardiomyocytes^18^, and hepatocytes^19^. The cytoplasmic sequestration of cyclin D1 is important to maintain the non-proliferative state as nuclear enforced expression using a nuclear-localized form of cyclin D1 forces the cell into a proliferative state^18^. Cyclin D1 has been identified in the cytoplasmic membrane^20–22^ and shown to bind and regulate the function of several cytoplasmic membrane-associated proteins including PACSIN II (Protein kinase C and Casein kinase Substrate In Neurons protein 2)^23^ also known as syndapin), Filamin A^24^ and paxillin^21^.

The association of cyclin D1 with cytoplasmic membrane proteins^21,23,24^ is consistent with prior studies demonstrating other components of the cell-cycle control apparatus are located in the cytoplasmic membrane including p27^Kip1^ and p16^INK4a^ ^20,25^. Although the physiological function of cytoplasmic membrane-associated cell-cycle components was previously not well understood, p16^INK4a^ and CDK6 colocalize in membrane ruffles of spreading cells and functioned upstream of αvβ3-dependent activation of PKC to regulate matrix-dependent cell migration^25^. Cyclin D1-deficient mouse embryo fibroblasts (MEFs) and mammary epithelial cells exhibit increased adhesion and decreased motility compared with wild-type MEFs^26–28^. Transduction of *cyclin D1*^*−/−*^ cells with a human or murine cyclin D1 cDNA, reversed this adhesive phenotype, promoting cell migration^26^. The induction of cell migration by cyclin D1 correlated with the reduction of Rho GTPase activity^26^. Mutational analysis demonstrated that cyclin D1 reduction of cellular adhesion and induction of cellular migration were independent of the pRB- and p160 coactivator-binding domains^26^. Cyclin E knockin of *cyclin D1*^*−/−*^ MEFs rescued the DNA synthetic defect of *cyclin D1*^*−/−*^ MEFs but did not rescue the migration defect^26^ suggesting the pRB binding of cyclins and the promigratory function may be dissociable.

Although cyclin D1 binds cytoplasmic membrane-associated proteins and correlative studies have suggested that cyclin D1 may promote cellular migration, no studies have selectively uncoupled the functional activity of the nuclear *vs*. cytoplasmic cyclin D1 pools. The current studies were conducted in order to determine the function of cyclin D1 when localized to either the cellular membrane or the nucleus.

## Results

### Cyclin D1 is located at the cytoplasmic membrane

**Fig. 1:**
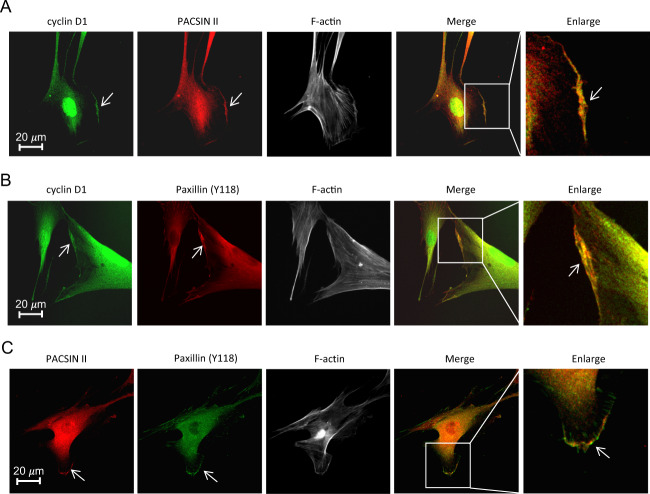
Cyclin D1 is located in the cytoplasmic membrane. **a** The human diploid fibroblast cell line (MRC-5) was stained for cyclin D1, PACSIN II, and F-actin. Merged images demonstrate the presence of cyclin D1 at the membrane (arrow), shown at high magnification in the right-side panel. Size bar is 20 μm. **b** Cyclin D1 co-staining with tyrosine phosphorylated Paxillin (Y118) and F-actin. Focal contacts are identified by the tyrosine phosphorylated Paxillin. **c** Co-staining of PACSIN II with tyrosine phosphorylated Paxillin (Y118) with merged staining shown by yellow arrows.

The endogenous cytoplasmic membrane-associated protein PACSIN II was shown to bind cyclin D1 in liver tissue^23^ and cyclin D1 bound to PACSIN II and paxillin (Pxn) in 3T3 cells^21,23^. In order to characterize the function of membrane-associated cyclin D1, studies were conducted in the human diploid fibroblast cell line (MRC-5) and human breast cancer samples. Using immunohistochemistry, endogenous cyclin D1 was identified at the MRC-5 cellular leading edge, in proximity with PACSIN II (Fig. 1a, Fig. S1). Paxillin (Pxn) is a structural and regulatory component of FAs and is also found along the cell membrane. Cyclin D1 was identified co-staining with tyrosine phosphorylated paxillin (Paxillin-P^Tyr118^ Fig. 1b, Fig. S2A). PACSIN II and tyrosine phosphorylated paxillin colocated at the leading edge Fig. 1c, Fig. S2B), consistent with prior studies conducted of the individual proteins in other cell types^21,23,24^.

Inflammatory breast cancer (IBC) is very aggressive breast cancer linked to poor prognosis. In order to assess the location of cyclin D1 in human breast cancer we compared membrane-associated cyclin D1 in patients with IBC and other breast cancers. Samples from 6 IBC patients and 17 non-IBC patients were stained for cyclin D1 and analyzed by a clinical pathologist (Fig. S3A, B). The subcellular distribution was assigned using the standard Aperio digital analysis algorithm for cell-membrane staining. The entire slide was scanned enabling analysis of >1000 cells per sample. Five of six IBCs stained for membrane associated cyclin D1, whereas only 2/17 non IBCs stained for membrane-associated cyclin D1. Cytoplasmic-membrane associated cyclin D1 was observed in 5 of 6 IBC patient samples and 2 of 17 non-IBC patient samples had membrane-associated cyclin D1 (Fig. S3). All 6 IBC and 16 of 17 non-IBC patients had nuclear localized cyclin D1. We next conducted immunofluorescent studies for cyclin D1 in non-IBC patients in order to provide more sensitive detection of membrane-associated cyclin D1. Costaining of cancer cells with a pan-cytokeratin antibody and underexposing the immunofluorescence (IF) signal provides an effective way of delineating the cellular boundaries, revealing tumor cores with clusters of cells displaying membrane-associated cyclin D1. In a tissue microarray of 50 ERα-positive breast cancers examined, membrane-associated cyclin D1 was detected in four cases (Fig. S3C–F).

### Cytoplasmic membrane-targeted cyclin D1 promotes transwell cellular migration and increases cellular migratory velocity

**Fig. 2:**
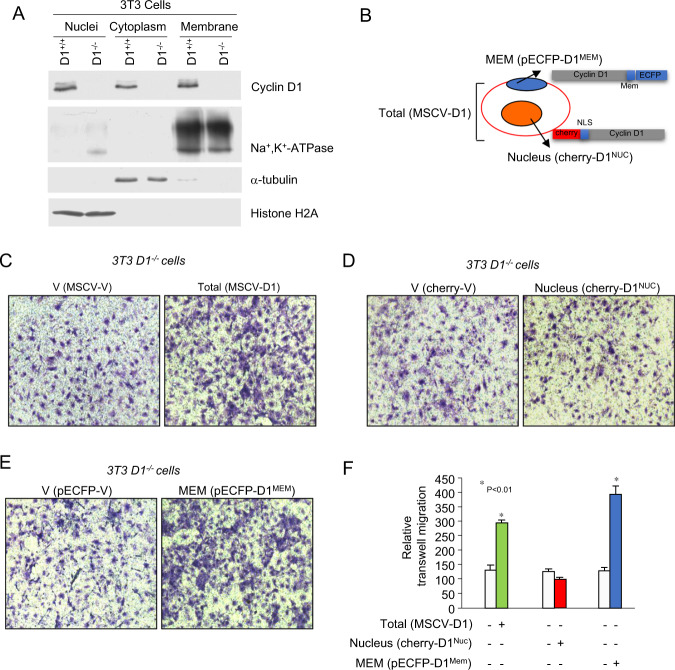
Cytoplasmic membrane-associated cyclin D1 promotes transwell migration. **a** Western blot analysis of 3T3 cells in which cellular subfractionation has been conducted. The subcellular fractions are characterized for enrichment of the nucleus (Histone H2A), cytoplasm (α-tubulin), or the cell membrane (Na^+^/K^+^ ATPase). **b** Schematic representation of the expression plasmids used for targeting cyclin D1 to the cytoplasmic membrane (pECFP-D1 (pECFP-D1^MEM^)) or nuclear targeted cyclin D1 (Cherry-D1, (Cherry-D1^NUC^)). **c** Transwell migration assays of *cyclin D1*^*−/−*^ 3T3 cells rescued with expression vectors encoding either cyclin D1^WT^ or **d** nuclear localized cyclin D1^NUC^ (cherry-D1^NUC^) or **e** membrane-associated cyclin D1^MEM^ (pECFP-D1^MEM^). **f** Data are shown as mean ± SEM for *N* = 5.

Cyclin D1 is known to promote cellular migration of fibroblasts and mammary epithelial cells^26,28^. In order to further characterize the molecular mechanisms by which cyclin D1 governs the induction of cellular migration we conducted subfractionation of nuclear and cytoplasmic cellular fractions from cyclin D1^WT^ *vs cyclin D1*^*−/−*^ 3T3 cells (Fig. 2a). Western blot analysis demonstrated enrichment of histone H2A in the nuclear fraction, α-tubulin in the cytoplasmic fraction and Na^+^/K^+^-ATPase in the membrane-associated fraction as previously described^29^. Cyclin D1 was identified in each of the subcellular fractions, consistent with prior studies conducted by confocal microscopy^21,23^. In order to determine the function of the cytoplasmic membrane-localized fraction of cyclin D1, *cyclin D1*^*−/−*^ 3T3 were transduced with a cyclin D1 expression vector encoding either cyclin D1^WT^, cyclin D1^NUC^, or cyclin D1^MEM^ (Fig. 2b) and functional analysis were conducted. Cherry-lacR-NLS-CD1^NUC^ which encodes a nuclear localized form of cyclin D1, was previously well characterized^30,31^. Cyclin D1 was cloned at the C-terminus of the Cherry-lacR-NLS vector^32,33^. For cyclin D1^MEM^ the cyclin D1 cDNA was cloned in frame to pECFP-Mem (Clonetech), which encodes a fusion protein consisting of the N-terminal 20 amino acids of neuromodulin, also called GAP-43, and a cyan fluorescent variant of the enhanced green fluorescent protein. The neuromodulin fragment contains a signal for posttranslational palmitoylation of cysteines 3 and 4 that targets ECFP to cellular membranes. Expression of ECFP-Mem in mammalian cells results in strong labeling of the plasma membrane and had been used to target proteins including ERα to the plasma membrane^34^. Using electroporation the transfection efficiency was >90%. Cyclin D1^WT^ enhanced transwell migration twofold (Fig. 2c, f), cyclin D1^NUC^ did not enhance transwell migration (Fig. 2d, f) and cyclin D1^MEM^ enhanced transwell migration threefold (Fig. 2e, f).

Transwell migration assays were next conducted in MCF-7 cells that were serum starved to reduce endogenous cyclin D1. Compared with the respective vector control transwell migration was enhanced 6.6-fold by cyclin D1^WT^, 2.2-fold by cyclin D1^NUC^ and 12.6-fold by cyclin D1^MEM^ (Fig. S4).

**Fig. 3:**
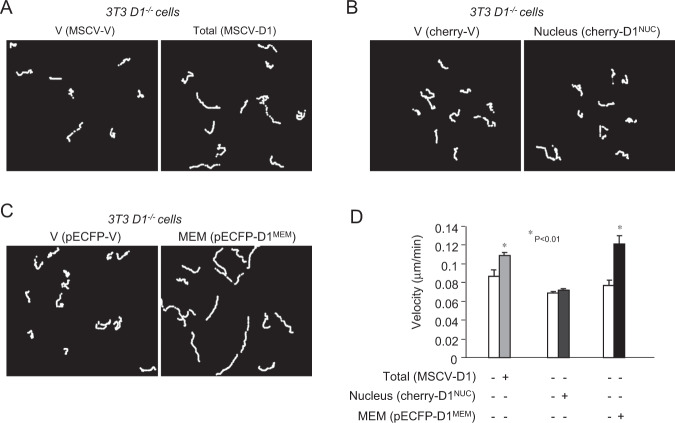
Membrane-associated cyclin D1 promotes cellular migratory velocity. **a** Time lapse videomicroscopy analysis was used to determine migration velocity of *cyclin D1*^*−/−*^ 3T3 cells rescued with expression encoding either cyclin D1^WT^ or **b** nuclear localized cyclin D1^NUC^ (cherry-D1^NUC^) or **c** membrane-associated cyclin D1^MEM^ (pECFP- D1^MEM^). **d** Data are shown as mean ± SEM for *N* = 20.

*Cyclin D1*-deficient fibroblasts show the same diameter size as wild-type cells, but attach and spread more rapidly after seeding on fibronectin-coated plates^26,28^. Herein, time lapse video microscopy demonstrated the induction of cellular velocity by cyclin D1^WT^ (Fig. 3a, d). Expression of Cyclin D1^MEM^, but not cyclin D1^NUC^, promoted cellular migratory velocity (Fig. 3b–d).

The subcellular distribution of cyclin D1^MEM^ and cyclin D1^NUC^, was further characterized using confocal microscopy (Fig. S5). Transfected cells were examined by confocal microscopy and by Z series reconstruction with the nucleus stained with Hoechst 33342. The cells expressing membrane-associated cyclin D1 showed green fluorescence predominantly at the cellular membrane (Fig. S5A, B), whereas the cyclin D1^NUC^ showed red fluorescence predominantly in the nucleus (Fig. S5C, D).

### Cytoplasmic membrane-targeted cyclin D1 augments DNA synthesis and contact independent growth

**Fig. 4:**
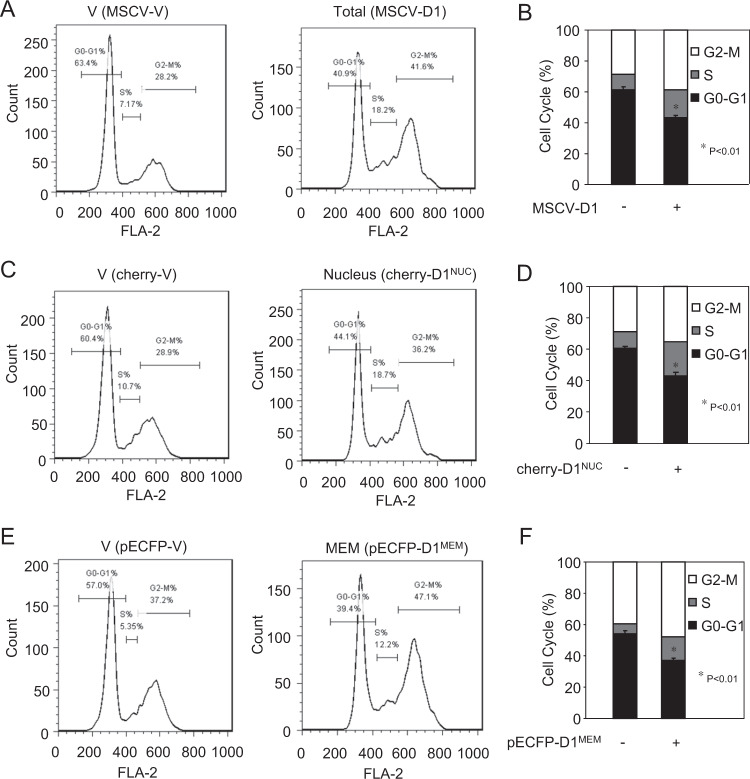
Membrane-associated cyclin D1 promotes S-phase entry. **a** Fluorescence activated cell sorting of *cyclin D1*^*−/−*^ 3T3 cells rescued with expression encoding either cyclin D1^WT^ or **c**, **d** nuclear localized cyclin D1 (cherry-CD1^NUC^) or **e**, **f** membrane localized cyclin D1 (pECFP-D1 ^MEM^). **b**, **d**, **f** Data for cell-cycle distribution are shown as mean ± SEM.

Reintroduction of cyclin D1 into *cyclin D1*^*−/−*^ fibroblasts may enhance DNA synthesis associated with a reduction in the proportion of cells in the G_0_/G_1_ phase of the cell cycle. In order to determine the capacity of membrane-targeted cyclin D1 to regular the cell-cycle distribution, fluorescence activated cell sorting (FACS) analysis was conducted. Comparison was made to the empty control vector because of the potential impact of fluorescent proteins on apoptotic and cell-cycle control proteins^35^. The distribution of cells in each phase of the cell cycle assessed by FACS demonstrated cyclin D1^WT^, cyclin D1^NUC^, and cyclin D1^MEM^ enhanced the proportion of cells in the DNA synthetic (S) phase with a doubling of the proportion of cells in S phase by cyclin D1^WT^ (7.2 vs. 18.2%) (Fig. 4a, b), an 80% increase in S phase by cyclin D1^NUC^ (10.7 vs. 18.7%) (Fig. 4c, d) and a doubling of the proportion of cells in S phase by cyclin D1^MEM^ (5.35 vs. 12.2%) (Fig. 4e, f).

**Fig. 5:**
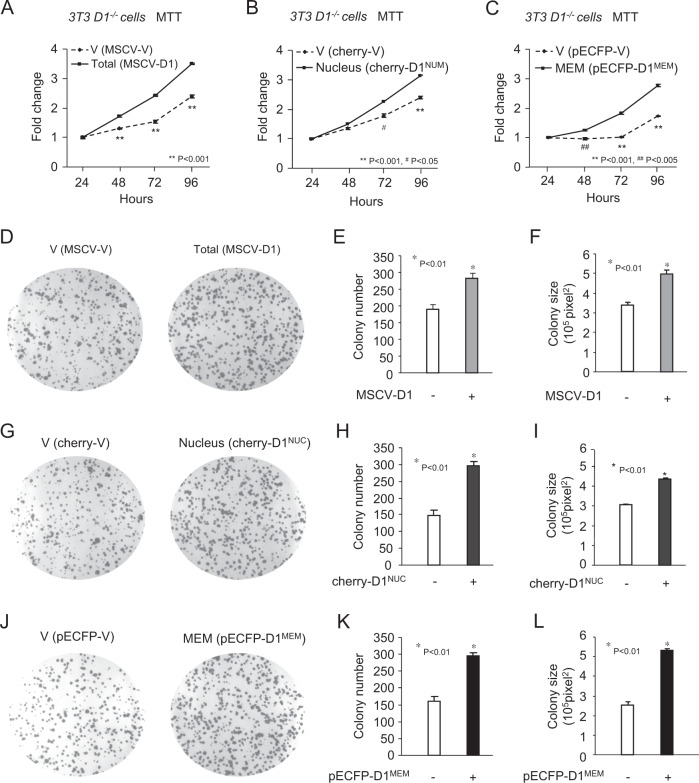
Membrane-associated cyclin D1 is sufficient for the induction of both the size and number of new colonies formed. **a** *Cyclin D1*^*−/−*^ 3T3 cells rescued with expression encoding either cyclin D1^WT^ or (nuclear localized cyclin D1 (cherry-D1^NUC^) or membrane associated cyclin D1 (pECFP-D1 ^MEM^) were analyzed for (**a**–**c**), cellular proliferation determined by the MTT assay or (**d**–**l**), colony formation assays. The data are shown for either the colony number or colony size as mean ± SEM for *N* = 5 separate experiments. Statistical analysis was conducted using the student *t* test, and the *P* value is shown in the figure.

Cellular proliferation was assessed by MTT activity with comparison made to the control vector. Cellular proliferation was increased by cyclin D1^WT^ (2.4 vs. 3.5-fold), cyclin D1^NUC^ (2.4 vs. 3.1-fold) and cyclin D1^MEM^ (1.6 vs. 2.7-fold) (Fig. 5a). Colony formation as an assay of contact-independent growth, showed an increase in both colony number and colony size with either cyclin D1^WT^, cyclin D1^NUC^ or cyclin D1^MEM^ (Fig. 5d–l) with a twofold increase in colony number and size with cyclin D1^MEM^ (Fig. 5j–l). In order to determine potential mechanisms by which cyclin D1^NUC^ and cyclin D1^MEM^ may induce proliferative signaling, we assessed the impact of signaling induced using downstream reporter target genes (Fig. S6). Consistent with prior studies, that cyclin D1 repressed the (AOX)3-LUC reporter gene^36^, herein both cyclin D1^NUC^ and cyclin D1^MEM^ repressed the (AOX)3-LUC reporter gene (Fig. S6). The immediate early gene c-*Fos*-LUC and *cyclin D1*-LUC were induced approximately twofold more by cyclin D1^MEM^ than by cyclin D1^NUC^ (Fig. S6). These studies show that cyclin D1^MEM^ activates immediate early gene c-*Fos* and *cyclin D1* transcription and suggest that cyclin D1^MEM^ may promote distinct signaling pathways to augment cellular growth.

### Cytoplasmic membrane-targeted cyclin D1 augments estrogen-dependent Akt kinase activation via K112

**Fig. 6:**
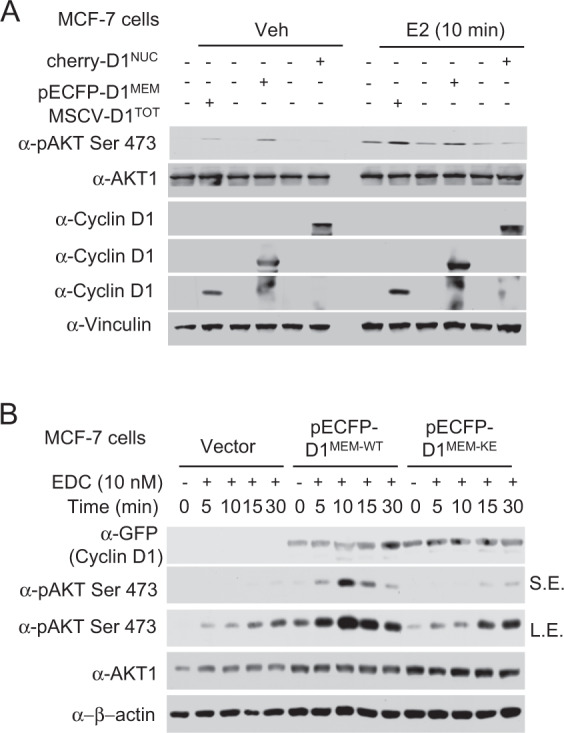
Membrane-associated cyclin D1 augments Akt signaling induced by estrogen. **a** Western blot analysis of MCF-7 cells transduced with expression vectors encoding cyclin D1 located to the nucleus (cherry-D1^NUC^), the cytoplasmic membrane (pECFP-D1^MEM^) or cyclin D1 expressed in both compartments (Total-MSCV-D1^TOT^). Cells were treated with E_2_ (10 nM) for 10 min. Vinculin is a protein loading control. **b** Cells transfected with expression plasmids encoding cytoplasmic membrane targeted cyclin D1^MEM^^-^^WT^ or cyclin D1^MEM^^-^^KE^ were treated with 17β estradiol dendrimer conjugate (EDC, 10 nM) for the time points indicated and Western blot conducted for Akt or a phosphorylated substrate (pAkt Ser 473). S.E. short exposure, L.E. long exposure.

The estrogen receptor α (ERα) is known to convey both genomic and extra genomic activities^37^. The extranuclear estrogen signaling pathway is thought to involve a membrane-associated ERα, which activates PI3-kinase and thereby Akt signaling^38^. Maximal activation of Akt requires phosphorylation on the carboxy-terminal site, S473, by mTORC2^39,40^. In recent studies, membrane-associated estrogen signaling was shown to occur via cyclin D1^32^. We investigated the impact of expressing cyclin D1 as either total, nuclear, or membrane-tethered forms of cyclin D1 (Fig. 6). The human breast cancer cell line (MCF-7) was transduced with expression vectors encoding cyclin D1 targeted to the nucleus (Cherry-CD1^NUC^), to the cytoplasmic membrane (PECFP-CD1^MEM^) or expressed in both cytoplasmic and nuclear compartments (MSCV-CD1^TOT^). Increased expression of cyclin D1 via an MSCV expression vector (cyclin D1^WT^), resulted in increased cyclin D1 abundance and increased phosphorylation of Akt1 at Ser473 compared with vector control (Fig. 6a, lanes 1 vs. 2). The ectopic expression of cyclin D1^MEM^ enhanced phosphorylation of Akt1 at Ser473 compared with vector control (Fig. 6a, lanes 3 vs. 4). Estradiol (E_2_) increased phosphorylation of Akt1 at Ser473 compared with vehicle control (Fig. 6a, lanes 7 vs. 1). The ectopic expression of cyclin D1^MEM^ increased E_2_-induced phosphorylation of Akt1 at Ser473 compared with vehicle control (Fig. 6a, lanes 10 vs. 4).

The extranuclear vs. nuclear E_2_-induced signaling pathways can be distinguished using 17β-estradiol linked to a dendrimer conjugate (EDC), which excludes estradiol from the nucleus^41,42^. In order to define the residues of cyclin D1 that participate in Akt activation, mutational analysis of cyclin D1 was conducted. Breast cancer epithelial cells (MCF-7 cells) were treated with either EDC or dendrimer control. Expression of a membrane-associated cyclin D1 under control of the MSCV promoter (cyclin D1^MEM^) induced phosphorylation of Akt1 at Serine473 (Fig. 6b, lanes 1 vs. 6). The addition of EDC to cyclin D1^MEM^ MCF-7 cells, augmented phosphorylation of Akt1 at Serine473 (Fig. 6b, lanes 6 vs. 7, 5 min S.E. (shorter exposure)). Mutation of cyclin D1 at K112 reduces CDK4/6 and p27^KIP1^ binding^43,44^. Expression of a membrane-tethered mutant of cyclin D1 at K112 (cyclin D1^MEM-KE)^) demonstrated an approximately 90% reduction in EDC-mediated induction of Akt1 Serine473 phosphorylation compared with empty vector control cells (Fig. 6b, lane 1 vs. 11; lanes 2 vs. 12).

### Cytoplasmic membrane-targeted cyclin D1 augments EDC-dependent Akt kinase activation at the cell membrane

**Fig. 7:**
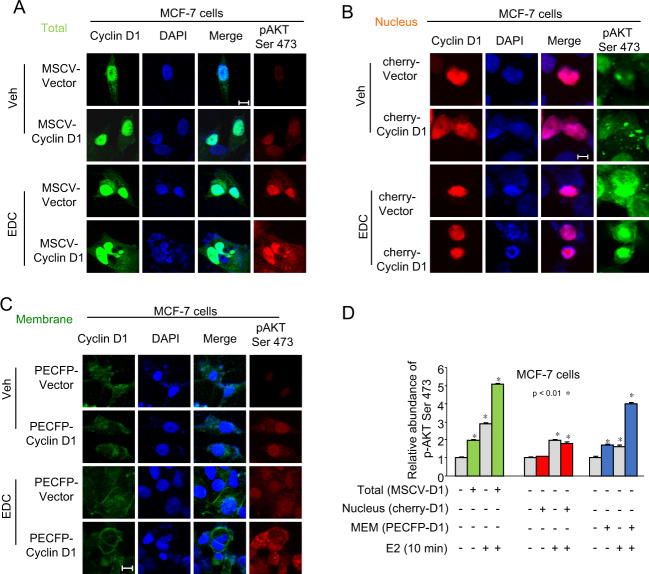
Membrane-associated cyclin D1 augments Akt1 Ser473 phosphorylation induced by extranuclear localized estradiol dendrimer (EDC). **a** Immunohistochemical staining for MCF-7 cells expressing either an expression vector for cyclin D1 tethered to the cytoplasmic membrane (PECFP-CD1^MEM^), **b** nuclear localized cyclin D1 (cherry-CD1^NUC^), or **c** or expressed in both compartments (cyclin D1 (Total-MSCV-CD1^TOT^)). Cells were treated with either EDC or vehicle control. DAPI is used as a nuclear stain. **d** The relative abundance of phosphorylated Akt (Serine 473) are shown for each of the treatment paradigms as mean ± SEM where *N* = 50 separate cells. EDC (10 nM) was used to treat the cells for 10 min.

We conducted IF to assess the relative abundance and subcellular distribution of Akt1 Serine 473 phosphorylation upon EDC treatment in cells transduced with the distinct located forms of cyclin D1. MCF-7 cells expressing the membrane-associated cyclin D1 (PECFP-CD1^MEM^) showed the characteristic enrichment of membranous GFP staining (Fig. 7a). Akt1 phosphorylated at Serine473 may be either nuclear or cytoplasmic, related to additional signaling partners^45^. In the vehicle treated cells, cyclin D1^MEM^ expression was associated with the induction of nuclear p-Ser473-Akt1. EDC treatment of vector control cells increased nuclear p-Ser473-Akt1. EDC treatment of cyclin D1^MEM^ transduced MCF-7 cells correlated with the induction of p-Ser473-Akt1, which was found to be in a cytoplasmic membranous distribution (Fig. 7a). In MCF-7 cells transduced with cyclin D1^NUC^, cyclin D1-RFP was located primarily in the nucleus. Nuclear localized cyclin D1 (cherry-CD1^NUC^) did not induce p-Ser473-Akt1 significantly (Fig. 7b). EDC treatment of MCF-7 cells augmented phosphorylation p-Ser473-Akt1, which was primarily nuclear in distribution (Fig. 7b). Careful quantitation evidenced that cyclin D1^NUC^ did not augment EDC-induced nuclear Akt1 Serine473 phosphorylation (Fig. 7b, d). MCF-7 cells transduced with cyclin D1^TOT^ showed nuclear, cytoplasmic, and membrane-associated cyclin D1, and an enhancement of EDC induced Akt1 Serine473 phosphorylation. p-Ser473-Akt1 was located in both the nucleus and membrane (Fig. 7c, d). Thus, the cytoplasmic membrane localized cyclin D1 (PECFP-CD1^MEM^), but not the nuclear localized form (cherry-CD1^NUC^), augmented Akt1 phosphorylation at Serine473.

### The immediate activation of Akt1 by insulin requires cyclin D1

Recent studies identified a dichromic fluorescent (DCF) dye substrate for cellular Akt1 activity^46^. The diserine DCF substrate was shown to serve as a specific substrate for Akt1, which can be used to quantitatively assess the enzyme’s activity in real time^46^. Insulin activation of cellular Akt phosphorylates a single serine residue of the diserine DCF substrate in a time dependent manner, resulting in a spectral shift that can be used to assess longitudinally the stimulation and reversibility of Akt1 activity. The dichromic dye LS456 is phosphorylated by Akt1, but not a variety of other kinases (including PKA, PKC, RSK1, P70S6K, and PI3K)^46^. The binding of insulin to its cell surface receptor stimulates phosphoinositide-3 kinase (PI3K), which then induces the second messenger, phosphotidylinositol-3, 4, 5-triphosphate (PIP3). PIP3 activates Akt and additional downstream effectors. As LS456 was shown to serve as a specific substrate for Akt1 in response to 150 nM insulin, we examined the kinetics of insulin-mediated activation of LS456 in *cyclin D1*^*−/−*^ MEF compared with wild-type MEFs. Insulin stimulation of Akt1 activity assessed by LS456 was delayed with reduced induction in *cyclin D1*^*−/−*^ cells compared with the cyclin D1^WT^ rescued cells (Fig. S7).

### Cyclin D1 restrains RhoA activity via K112

**Fig. 8:**
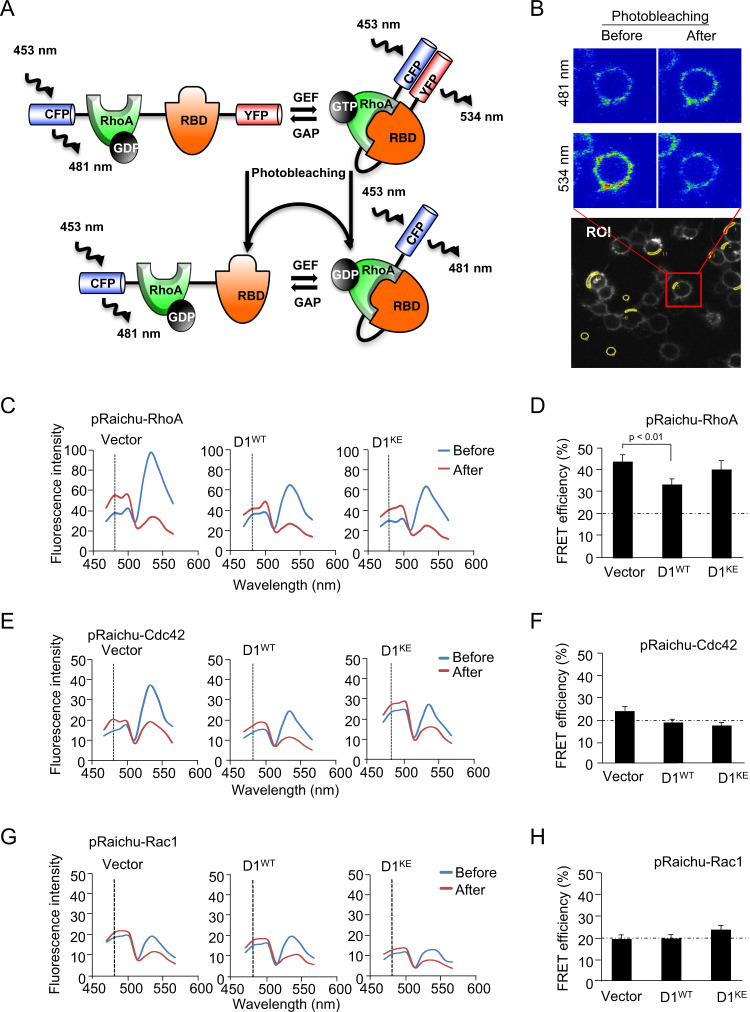
Cyclin D1 restrains RhoA activity assessed by FRET. **a** Schematic representations of pRaichu-RhoA (RhoA (aa 1–189) bound to GDP or GTP. YFP and CFP denote a yellow and cyan-emitting mutant of GFP, respectively. RhoA-binding domain (RBD) indicates the RBD of the effector protein. **b** Representative image of cellular membrane associated emission at 534 and 481 nm before and after photobleaching. **c** Emission spectra of pRaichu-RhoA expressed in HEK-293T cells at an excitation wavelength of 453 nm (left) with fluorescence intensity mapped before (blue) and after (red) photobleaching. Cells were co-transfected with expression vectors for cyclin D1 or the cyclin D1 K112 mutant of cyclin D1 (cyclin D1^KE^). **d** FRET efficiency is shown calculated for *N* > 8 separate cells. Data are shown for **e**, **f** of pRaichu-Cdc42 and **g**, **h** of pRaichu-Rac1.

In the current studies, cytoplasmic membrane-tethered cyclin D1 augmented cellular migratory velocity and estrogen-dependent induction of Akt1 Ser473 phosphorylation. In prior studies cyclin D1 rescue of *cyclin D1*^*−/−*^ MEFs reduced RhoA activity^26^. Although these prior studies suggested that cyclin D1 may augment cellular migration by restraining RhoA activity, Rac1 and Cdc42 can also participate in cellular migration^47^. In order to examine the functional interactions with cyclin D1 and Rho GTPases we deployed the FRET based fluorescent probes for RhoA, Rac, and Cdc42 (Fig. 8a). pRaichu-RhoA consists of a truncated RhoA (aa 1–189), the RhoA-binding domain (RBD) and the FRET pair of CFP and YFP. When RhoA binds to GTP, and thereby the RBD, RhoA recruits CFP in close proximity to YFP, thereby increasing the FRET activity between CFP and YFP. We examined the functional interaction between cyclin D1 and RhoA using FRET. The image from a typical FRET experiment was shown in Fig. 8b. Cells were co-transfected with pRaichu-RhoA and either cyclin D1^WT^, cyclin D1^KE^, or their corresponding vector control. Spectral images in 10 channels from 470 to 566 nm with excitation at 458 nm were simultaneously recorded. YFP was inactivated by photobleaching with a 514 nm laser at 100% power output (Fig. 8b). The emission spectra within the ROI increased in the CFP signal at 481 nm after photobleaching with YFP which has an emission peak at 534 nm (Fig. 8c). FRET efficiency was used to quantitatively compare the difference in RhoA activity among the cells. (FRET efficiency was defined as (*F*_*B*_ − *F*_0_)/*F*_*B*_ × 100%, where *F*_*B*_ is the intensity of the donor (CFP) after photobleaching and *F*_0_ is the intensity of the donor before photobleaching, see “Methods”). FRET efficiency was reduced 40% by cyclin D1^WT^ but was not significantly reduced by expression of the cyclin D1^KE^ (Fig. 8d). Similar analysis of FRET for the related Rho family members, Rac1 and Cdc42, failed to elicit changes in FRET efficiency upon re-expression of cyclin D1 wild type. By using FRET, we extend prior studies demonstrating cyclin D1 reduces Rho GTPase activity^26^, to define the interaction of cyclin D1 occurs with RhoA, not Rac or Cdc42, and demonstrate the residue K112 of cyclin D1 is required for interaction with RhoA.

## Discussion

The well characterized nuclear functions of cyclin D1 include firstly, serving as the regulatory subunit of a holoenzyme that phosphorylates the pRB protein, and secondly serving as part of a transcriptional regulatory complex that drives proliferative gene expression^48^. Consistent with previous studies, that either identified cyclin D1 associated with the cytoplasmic membrane or cytoplasmic membrane proteins^20–24^, the current studies identified cyclin D1 colocalized with PACSIN II and paxillin PTyr118 at the cytoplasmic membrane. The current studies extend our understanding of cyclin D1 through characterizing the function and signaling pathways regulated by cyclin D1 at the cytoplasmic membrane vs. the nucleus. Firstly, herein membrane-associated cyclin D1 augmented transwell migration and enhanced the velocity of cellular migration. In contrast, the nuclear-localized form of cyclin D1 neither enhanced cellular migratory velocity nor induced transwell migration in 3T3 cells. These studies are consistent with previous findings that cyclin D1 promotes migration^21,26,28,43,44^, but extend these findings by demonstrating that it is the membrane-associated form of cyclin D1 that mediates this function. Secondly, these studies show both nuclear and membrane-associated cyclin D1 augment cellular DNA synthesis, cellular proliferation, and contact-independent growth. Thirdly, these studies demonstrate that cyclin D1 tethered to the cytoplasmic membrane induces Akt signaling, characterized by the induction of Akt1 Ser473 phosphorylation. Furthermore, membrane-associated cyclin D1 augmented a physiological function of estrogen, to induce Akt1 Ser473 phosphorylation. Fourthly, as activity of Rho GTPase at the cellular membrane may inhibit cellular adhesion and migration and restrain Akt activity^49^, we examined and defined a role for cyclin D1 to inhibit Rho activity. Collectively these studies define a novel function for cytoplasmic membrane associated cyclin D1 that may augment aberrant growth control and cellular invasion.

Prior studies had shown the induction of cellular migration by cyclin D1^21,26,28,43,44^. *Cyclin D1*^*−/−*^ cells show a more spread morphology than the corresponding wild type and display an increased number of focal adhesions (FAs) with higher levels of tyrosine-phosphorylated paxillin^21,26,28,43,44^. Herein, using *cyclin D1*^*−/−*^ cells, we demonstrated the membrane-associated pool of cyclin D1 is sufficient to augment transwell migration. We identified cyclin D1 at the plasma membrane in inflammatory breast cancer, and cyclin D1 colocalized to the cytoplasmic membrane with PACSIN II and Paxillin (Y118) in MRC-5 cells. Cyclin D1 was previously shown by mass spectrometry to bind the membrane-associated proteins PACSIN II^23^, Filamin A^24^, Paxillin^21^, and several additional proteins^50^. PACSIN II is involved in cell spreading^51^, as well as endocytosis of cell–surface receptors like the EGF receptor^52^ and in caveolae-mediated endocytosis^53,54^. In view of clinical analyses showing a correlation between total cyclin D1 expression and tumor invasiveness and metastasis^12–15^, our studies suggest further studies assessing membrane-associated cyclin D1 may be warranted.

Herein, cytoplasmic membrane-associated cyclin D1 augmented phosphorylation of Akt1 at Ser473. Akt, also known as Protein Kinase B, promotes cellular survival, proliferation, growth, and migration^55^. Akt hyperactivation contributes to human cancer correlating with poor prognosis and therapy resistance and genetic deletion demonstrated *Akt1* is required for ErbB2-induced breast cancer progression and tumor metastases in vivo^56^. Herein the acute nongenomic E_2_ activation of Akt1, was augmented by the membrane-associated cyclin D1 pool. Estradiol acutely activates Akt^57,58^ in part through the association of ERα at the plasma membrane associated with the p85 regulatory subunit of PI3-kinase and other proteins including the scaffold protein caveolin-1, G proteins, Src kinase, Ras, and Shc^57,59–61^. ERα regulates nuclear gene expression via genomic and extranuclear non-genomic signals^37,59^. Extranuclear pools of ERα reside in the plasma membranes^62^ and the ability to distinguish nuclear from extranuclear ERα signaling has been enabled through the generation of a 17β-estradiol dendrimer conjugate (EDC) which is localized to the extranuclear compartment^41,42^. Herein, using nuclear excluded E_2_ dendrimers, cyclin D1 was shown to participate in the acute non-genomic E_2_ response. Genetic deletion studies in the mouse demonstrated E_2_-dependent induction of genes governing growth factors, growth factor receptor and promigratory processes in the mammary gland requires *cyclin D1*^63^. The biological effects of estrogen, are critically dependent upon cyclin D1 in vivo^63,64^, with the current studies suggesting an important component is mediated via membrane-associated cyclin D1.

RhoA, Rac1, and Cdc42 are the best characterized members of the Rho GTPase branch of the Ras superfamily and are known to regulate cellular morphology and migration^47^. In the current studies, cyclin D1 restrained RhoA activity, requiring K112. *Cyclin D1*^*−/−*^ cells have increased RhoA activity, increased ROCK II kinase and increased LIM kinase activation (threonine 505/508). LIM kinase phosphorylation at threonine 505/508 in turn phosphorylates the actin-depolymerizing protein cofilin at serine 3 and MLC2 at Thr18/Ser19^26^. Herein, FRET analysis evidenced cyclin D1 restrained Rho GTPase activity. In contrast, neither Rac-GTPase nor Cdc42 activity was influenced by cyclin D1. The reduction in RhoA GTPase FRET by cyclin D1 was abolished by mutation of cyclin D1 residue K112. Cyclin D1 participates in multiple functions via K112 including CDK4/6-mediated pRB phosphorylation^65^ and binding to p27^KIP1^^44^. Rho GTPase is an important modulator of ERα activity^66,67^, and E_2_ enhances ERα association with the p85 subunit of PI3 kinase thereby inducing Akt phosphorylation^57^. An increase in ERα/PI3K interactions in patient-derived xenografts (PDXs) correlates with acquired resistance to tamoxifen^68^. RhoA represses Akt Ser473 phosphorylation^49^ and the repression of RhoA activity by cyclin D1 may have contributed to the induction of pAkt1–Ser473. The role of cyclin D1 in restraining RhoA, thereby inducing ERα activity and tamoxifen resistance, warrants further investigation.

Several lines of evidence support the importance of cyclin D1 nuclear location in aberrant growth including elegant studies showing that a mutant of cyclin D1 (D1T286A), that is defective in phosphorylation-mediated nuclear export, induces cell transformation in cell culture assays and triggers B-cell lymphoma in a mouse model of mantle cell lymphoma^69,70^. Furthermore, transgenic mice that overexpress the identical mutant cyclin D1 driven by the MMTV promoter (MMTV-D1T286A) developed mammary adenocarcinoma with a shorter latency relative to mice over-expressing the wild-type cyclin D1 (MMTV-D1)^71^. That said, the current studies suggest that in addition to the nuclear function of cyclin D1, a membrane-associated pool of cyclin D1 contributes to cellular migration, induction of Akt1 activity and the induction of a signaling pathway, defined through transcriptional reporters, that activates the immediate early gene c-*Fos* and *cyclin D1*. c-*Fos* is a target of Akt1 induction and Fos family members induce cell-cycle entry though the induction of cyclin D1^72^, suggesting a mechanism by which membrane associated cyclin D1 may augment cellular growth. The major adjuvant therapy for the ~70% of ERα expressing human breast cancer involves anti-estrogen therapy. The ERα/PI3K/Akt complex pathway is hyperactivated in aggressive breast tumors^73^. The non-genomic actions of E_2_/ERα, mediated via cytoplasmic membrane-associated cyclin D1, may provide an important additional target^58^. As membrane-associated cyclin D1 augments activity of the ERα/PI3K/Akt complex pathway, the cytoplasmic membrane pool of cyclin D1 may be a new target for ERα expressing breast cancer treatments^74,75^.

## Materials and methods

A detailed description is provided in the Supplementary Materials.

### Plasmids and tissue culture

The *cyclin D1*^*+/+*^ and *cyclin D1*^*−/−*^ MEFs^10^ were prepared as described previously^76^.

### Transwell migration

The assessment of transwell migration^77^, migratory velocity, and migratory distance^26^ were conducted as previously described.

### Fluorescence resonance energy transfer (FRET) imaging

HEK293T cells, co-transfected with 3×FLAG vector, cyclin D1 wild-type or cyclin D1^KE^ mutant and FRET reporters (pRaichu-RhoA, pRaichu-Cdc42 or pRaichu-Rac1^78,79^), were cultured in a four-well chamber and imaged using a Zeiss laser-scanning microscope, LSM510META, with a 40× oil immersion Doc Plan-Neofluar lens objective (numerical aperture of 1.3). To detect FRET between CFP and YFP, we used time-lapse and lambda stack acquisition linked with the photobleaching command^80^.

### Immunostaining

IF staining and confocal microscopy of cultured cells was conducted as described previously^77^. Chromogen immunostaining of human breast cancer samples was conducted on the breast tissue with the Ventana Benchmark autostainer using deintified archival tissue which are exempt from review by the Thomas Jefferson University Institutional Review Board. Fluorescence-based immunohistochemistry for cyclin D1 multiplexed with pan-cytokeratin and DAPI counterstain was performed as previously described^81–83^ on a tissue microarray containing cores of 50 de-identified ER-positive breast cancer specimens provided by the Medical College of Wisconsin Tissue Bank under IRB-approved protocol.

### Live cell Akt activity monitoring

Live cell imaging studies were conducted as described^46^.

## Supplementary information

Supplemental Methods and Figure Legends

Supplemental Figures

## References

[1] Hanahan D, Weinberg RA (2011). Hallmarks of cancer: the next generation. Cell.

[2] Hassaan SH (2019). Assessment of cognitive functions and psychiatric symptoms in hepatitis C patients receiving pegylated interferon alpha and ribavirin: a prospective cohort study. Int J. Psychiatry Med..

[3] Sherr CJ (1994). G1 phase progression: cycling on cue. Cell.

[4] Hinds PW, Dowdy SF, Eaton EN, Arnold A, Weinberg RA (1994). Function of a human cyclin gene as an oncogene. Proc. Natl Acad. Sci. USA.

[5] Lee RJ (2000). Cyclin D1 is required for transformation by activated Neu and is induced through an E2F-dependent signaling pathway. Mol. Cell Biol..

[6] Yu B, Lane ME, Pestell RG, Albanese C, Wadler S (2000). Downregulation of cyclin D1 alters cdk 4- and cdk 2-specific phosphorylation of retinoblastoma protein. Mol. Cell Biol. Res. Commun..

[7] Hulit J (2004). Cyclin D1 genetic heterozygosity regulates colonic epithelial cell differentiation and tumor number in ApcMin mice. Mol. Cell Biol..

[8] Robles AI (1998). Reduced skin tumor development in cyclin D1-deficient mice highlights the oncogenic ras pathway in vivo. Genes Dev..

[9] Sicinski P (1995). Cyclin D1 provides a link between development and oncogenesis in the retina and breast. Cell.

[10] Casimiro MC (2012). ChIP sequencing of cyclin D1 reveals a transcriptional role in chromosomal instability in mice. J. Clin. Investig..

[11] Wang TC (1994). Mammary hyperplasia and carcinoma in MMTV-cyclin D1 transgenic mice. Nature.

[12] Drobnjak M, Osman I, Scher HI, Fazzari M, Cordon-Cardo C (2000). Overexpression of cyclin D1 is associated with metastatic prostate cancer to bone. Clin. Cancer Res..

[13] Hou X (2016). Cyclin D1 expression predicts postoperative distant metastasis and survival in resectable esophageal squamous cell carcinoma. Oncotarget.

[14] Huang H, Hu YD, Li N, Zhu Y (2009). Inhibition of tumor growth and metastasis by non-small cell lung cancer cells transfected with cyclin D1-targeted siRNA. Oligonucleotides.

[15] Noorlag R (2017). Amplification and protein overexpression of cyclin D1: predictor of occult nodal metastasis in early oral cancer. Head Neck.

[16] Borlakoglu JT, Stegeman J, Dils RR (1991). Induction of hepatic cytochrome P-450IA1 in pigeons treated in vivo with Aroclor 1254, a commercial mixture of polychlorinated biphenyls (PCBs). Comp. Biochem. Physiol. C.

[17] Sumrejkanchanakij P, Tamamori-Adachi M, Matsunaga Y, Eto K, Ikeda MA (2003). Role of cyclin D1 cytoplasmic sequestration in the survival of postmitotic neurons. Oncogene.

[18] Tamamori-Adachi M (2003). Critical role of cyclin D1 nuclear import in cardiomyocyte proliferation. Circ. Res..

[19] Jaumot M, Estanyol JM, Serratosa J, Agell N, Bachs O (1999). Activation of cdk4 and cdk2 during rat liver regeneration is associated with intranuclear rearrangements of cyclin-cdk complexes. Hepatology.

[20] Alhaja E (2004). Anti-migratory and anti-angiogenic effect of p16: a novel localization at membrane ruffles and lamellipodia in endothelial cells. Angiogenesis.

[21] Fuste NP (2016). Cytoplasmic cyclin D1 regulates cell invasion and metastasis through the phosphorylation of paxillin. Nat. Commun..

[22] Nebot-Cegarra J, Domenech-Mateu JM (1989). Association of tracheoesophageal anomalies with visceral and parietal malformations in a human embryo (Carnegie stage 21). Teratology.

[23] Meng H (2011). PACSIN 2 represses cellular migration through direct association with cyclin D1 but not its alternate splice form cyclin D1b. Cell Cycle.

[24] Zhong Z (2010). Cyclin D1/cyclin-dependent kinase 4 interacts with filamin A and affects the migration and invasion potential of breast cancer cells. Cancer Res..

[25] Fahraeus R, Lane DP (1999). The p16(INK4a) tumour suppressor protein inhibits alphavbeta3 integrin-mediated cell spreading on vitronectin by blocking PKC-dependent localization of alphavbeta3 to focal contacts. EMBO J..

[26] Li Z (2006). Cyclin D1 regulates cellular migration through the inhibition of thrombospondin 1 and ROCK signaling. Mol. Cell Biol..

[27] Li Z, Wang C, Prendergast GC, Pestell RG (2006). Cyclin D1 functions in cell migration. Cell Cycle.

[28] Neumeister P (2003). Cyclin D1 governs adhesion and motility of macrophages. Mol. Biol. Cell.

[29] Dunbar LA, Caplan MJ (2001). Ion pumps in polarized cells: sorting and regulation of the Na+, K+- and H+, K+-ATPases. J. Biol. Chem..

[30] Casimiro MC (2016). Cyclin D1 promotes androgen-dependent DNA damage repair in prostate cancer cells. Cancer Res.

[31] Li Z (2010). Alternative cyclin D1 splice forms differentially regulate the DNA damage response. Cancer Res..

[32] Li Z (2014). Cyclin D1 integrates estrogen-mediated DNA damage repair signaling. Cancer Res..

[33] Soutoglou E, Misteli T (2008). Activation of the cellular DNA damage response in the absence of DNA lesions. Science.

[34] Razandi M (2003). Identification of a structural determinant necessary for the localization and function of estrogen receptor alpha at the plasma membrane. Mol. Cell Biol..

[35] Coumans JV (2014). Green fluorescent protein expression triggers proteome changes in breast cancer cells. Exp. Cell Res..

[36] Wang C (2003). Cyclin D1 repression of peroxisome proliferator-activated receptor gamma expression and transactivation. Mol. Cell Biol..

[37] Levin ER (2011). Minireview: extranuclear steroid receptors: roles in modulation of cell functions. Mol. Endocrinol..

[38] Di Sante G, Di Rocco A, Pupo C, Casimiro MC, Pestell RG (2017). Hormone-induced DNA damage response and repair mediated by cyclin D1 in breast and prostate cancer. Oncotarget.

[39] Dibble CC, Cantley LC (2015). Regulation of mTORC1 by PI3K signaling. Trends Cell Biol..

[40] Hresko RC, Mueckler M (2005). mTOR.RICTOR is the Ser473 kinase for Akt/protein kinase B in 3T3-L1 adipocytes. J. Biol. Chem..

[41] Harrington WR (2006). Estrogen dendrimer conjugates that preferentially activate extranuclear, nongenomic versus genomic pathways of estrogen action. Mol. Endocrinol..

[42] Madak-Erdogan Z (2008). Nuclear and extranuclear pathway inputs in the regulation of global gene expression by estrogen receptors. Mol. Endocrinol..

[43] Li Z (2006). Cyclin D1 induction of cellular migration requires p27(KIP1). Cancer Res..

[44] Li Z (2008). Alternate cyclin D1 mRNA splicing modulates p27KIP1 binding and cell migration. J. Biol. Chem..

[45] Martelli AM (2012). The emerging multiple roles of nuclear Akt. Biochim. Biophys. Acta.

[46] Shen D (2013). Dual fluorescent molecular substrates selectively report the activation, sustainability and reversibility of cellular PKB/Akt activity. Sci. Rep..

[47] Lawson CD, Ridley AJ (2018). Rho GTPase signaling complexes in cell migration and invasion. J. Cell Biol..

[48] Pestell RG (2013). New roles of cyclin D1. Am. J. Pathol..

[49] Gordon BS (2014). RhoA modulates signaling through the mechanistic target of rapamycin complex 1 (mTORC1) in mammalian cells. Cell Signal..

[50] Jirawatnotai S (2011). A function for cyclin D1 in DNA repair uncovered by protein interactome analyses in human cancers. Nature.

[51] de Kreuk BJ (2011). The F-BAR domain protein PACSIN2 associates with Rac1 and regulates cell spreading and migration. J. Cell Sci..

[52] de Kreuk BJ, Anthony EC, Geerts D, Hordijk PL (2012). The F-BAR protein PACSIN2 regulates epidermal growth factor receptor internalization. J. Biol. Chem..

[53] Giridharan SS, Cai B, Vitale N, Naslavsky N, Caplan S (2013). Cooperation of MICAL-L1, syndapin2, and phosphatidic acid in tubular recycling endosome biogenesis. Mol. Biol. Cell.

[54] Senju Y, Itoh Y, Takano K, Hamada S, Suetsugu S (2011). Essential role of PACSIN2/syndapin-II in caveolae membrane sculpting. J. Cell Sci..

[55] Hers I, Vincent EE, Tavare JM (2011). Akt signalling in health and disease. Cell Signal..

[56] Ju X (2007). Akt1 governs breast cancer progression in vivo. Proc. Natl Acad. Sci. USA.

[57] Castoria G (2001). PI3-kinase in concert with Src promotes the S-phase entry of oestradiol-stimulated MCF-7 cells. EMBO J..

[58] Pedram A, Razandi M, Evinger AJ, Lee E, Levin ER (2009). Estrogen inhibits ATR signaling to cell cycle checkpoints and DNA repair. Mol. Biol. Cell.

[59] Bjornstrom L, Sjoberg M (2005). Mechanisms of estrogen receptor signaling: convergence of genomic and nongenomic actions on target genes. Mol. Endocrinol..

[60] Simoncini T (2000). Interaction of oestrogen receptor with the regulatory subunit of phosphatidylinositol-3-OH kinase. Nature.

[61] Song RX, Zhang Z, Santen RJ (2005). Estrogen rapid action via protein complex formation involving ERalpha and Src. Trends Endocrinol. Metab..

[62] Levin ER, Pietras RJ (2008). Estrogen receptors outside the nucleus in breast cancer. Breast Cancer Res. Treat..

[63] Casimiro MC (2013). Cyclin D1 determines estrogen signaling in the mammary gland in vivo. Mol. Endocrinol..

[64] Body S (2017). Cytoplasmic cyclin D1 controls the migration and invasiveness of mantle lymphoma cells. Sci. Rep..

[65] Baker GL, Landis MW, Hinds PW (2005). Multiple functions of D-type cyclins can antagonize pRb-mediated suppression of proliferation. Cell Cycle.

[66] Su LF, Knoblauch R, Garabedian MJ (2001). Rho GTPases as modulators of the estrogen receptor transcriptional response. J. Biol. Chem..

[67] Takahashi K (2011). Estrogen induces neurite outgrowth via Rho family GTPases in neuroblastoma cells. Mol. Cell Neurosci..

[68] Poulard C (2019). Oestrogen non-genomic signalling is activated in tamoxifen-resistant breast cancer. Int J. Mol. Sci..

[69] Alt JR, Cleveland JL, Hannink M, Diehl JA (2000). Phosphorylation-dependent regulation of cyclin D1 nuclear export and cyclin D1-dependent cellular transformation. Genes Dev..

[70] Gladden AB, Woolery R, Aggarwal P, Wasik MA, Diehl JA (2006). Expression of constitutively nuclear cyclin D1 in murine lymphocytes induces B-cell lymphoma. Oncogene.

[71] Lin DI (2008). Disruption of cyclin D1 nuclear export and proteolysis accelerates mammary carcinogenesis. Oncogene.

[72] Brown JR (1998). Fos family members induce cell cycle entry by activating cyclin D1. Mol. Cell Biol..

[73] Soderberg O (2006). Direct observation of individual endogenous protein complexes in situ by proximity ligation. Nat. Methods.

[74] Poulard C (2012). Activation of rapid oestrogen signalling in aggressive human breast cancers. EMBO Mol. Med..

[75] Poulard C, Rambaud J, Le Romancer M, Corbo L (2014). Proximity ligation assay to detect and localize the interactions of ERalpha with PI3-K and Src in breast cancer cells and tumor samples. Methods Mol. Biol..

[76] Albanese C (1999). Activation of the *cyclin D1* gene by the E1A-associated protein p300 through AP-1 inhibits cellular apoptosis. J. Biol. Chem..

[77] Jiao X (2008). Disruption of c-Jun reduces cellular migration and invasion through inhibition of c-Src and hyperactivation of ROCK II kinase. Mol. Biol. Cell.

[78] Lam AJ (2012). Improving FRET dynamic range with bright green and red fluorescent proteins. Nat. Methods.

[79] Yoshizaki H (2003). Activity of Rho-family GTPases during cell division as visualized with FRET-based probes. J. Cell Biol..

[80] Jiao X, Zhang N, Xu X, Oppenheim JJ, Jin T (2005). Ligand-induced partitioning of human CXCR1 chemokine receptors with lipid raft microenvironments facilitates G-protein-dependent signaling. Mol. Cell Biol..

[81] Jiao X (2018). CCR5 governs DNA damage repair and breast cancer stem cell expansion. Cancer Res..

[82] Peck AR (2016). Validation of tumor protein marker quantification by two independent automated immunofluorescence image analysis platforms. Mod. Pathol..

[83] Pestell TG (2017). Stromal cyclin D1 promotes heterotypic immune signaling and breast cancer growth. Oncotarget.

