# Supplementary material for: The membrane-associated form of cyclin D1 enhances cellular invasion
Source: Oncogenesis. 2020 Sep 18;9(9):83. doi: 10.1038/s41389-020-00266-y (PMC7501870; doi:10.1038/s41389-020-00266-y)
Supplement: Supplementary file 1 — Supplemental Methods and Figure Legends [file 41389_2020_266_MOESM1_ESM.docx]

**The membrane-associated form of cyclin D1 enhances cellular invasion.**

Ke Chen^3**^, Xuanmao Jiao^1**^, Anthony Ashton^1^, Agnese Di Rocco^1^, Timothy G. Pestell^3^, Yunguang Sun^9^, Jun Zhao^1^, Mathew C. Casimiro^1^, Zhiping Li^1^, Michael P. Lisanti^4^, Peter A. McCue^5^, Duanwen Shen^6^, Samuel Achilefu^6,7,8^, Hallgeir Rui^9^, Richard G. Pestell^1,2*^

**Supplemental MATERIALS AND METHOD**S

**Plasmids and Tissue Culture.** The *cyclin D1^+/+^* and *cyclin D1^-/-^* MEFs ^2^ were prepared as described previously ^1^. The MCF-7 cell line, HEK293T and MRC-5 diploid human cells were from the American Type Culture Collection (Manassas, VA). All cells were maintained in Dulbecco’s Modified Eagle’s Medium (DMEM) containing penicillin and streptomycin (100 mg of each/liter) and supplemented with 10% fetal bovine serum (FBS). The cyclin D1 cDNA was cloned in frame to pECFP-Mem (Clonetech), which encodes a fusion protein consisting of the N-terminal 20 amino acids of neuromodulin, also called GAP-43, and a cyan fluorescent variant of the enhanced green fluorescent protein (EGFP). The neuromodulin fragment contains a signal for posttranslational palmitoylation of cysteines 3 and 4 that targets ECFP to cellular membranes. Expression of ECFP-Mem in mammalian cells results in strong labeling of the plasma membrane and had been used to target proteins including ERα to the plasma membrane ^13^. Cherry-lacR-NLS-CD1^NUC^ which encodes a nuclear localized form of cyclin D1, was previously well characterized ^3, 9^. Cyclin D1 was cloned at the C-terminus of the Cherry-lacR-NLS vector ^15^ in the KpnI/XmaI sites. Primers were used as the following: Cyclin D1 forward: cggggtaccgaacaccagctcctgtgct; Cyclin D1a reverse: tccccccgggtcagatgtccacgtcccgca. All plasmid DNA constructs were verified by sequencing. The MCF-7 or *cyclin D1^-/-^* 3T3 cells were co-transfected with expression plasmids for cyclin D1 (P-ECFP-CD1^MEM^, Cherry-lacR-NLS-CD1^NUC^ and MSCV-CD1^TOT^) using Lipofectamine 2000 (Qiagen, Valencia, CA). The AOX3-LUC ^16^, the cyclin D1-LUC (-1745 CD1-LUC) and c-Fos-LUC and the methods of co-transfection with cyclin D1 expression vectors was previously described ^12^.

In the experiments with E_2_ or 17β estradiol (E_2)_-dendrimer (EDC), MCF-7 cells were cultured in phenol red-free DMEM with 10% charcoal/dextran–treated FBS and 2 mM glutamine for 24 hrs. 24 hrs post transfection the cells were treated with doses of E_2_ (0.1 nM, 1 nM, and 10 nM), EDC (10 nM) or vehicle (ethanol) control.

**Cell Proliferation Assays.** Cells were seeded into 96 well plates in normal growth medium, and cell growth was measured daily by MTT assays using 3-(4, 5-dimethylthiazol-2-yl)-2, 5-diphenyltetrazolium bromide.

**Transwell Migration.** Transwell migration assays were conducted as previously described ^5^. GFP-positive cells were seeded on 8-μm-pore-size Transwell filter insert (Costar) coated with 10 μg/ml fibronectin (Sigma, St. Louis, MO). After 16 h of incubation at 37°C and 5% CO_2_, cells adherent to the upper surface of the filter were removed using a cotton applicator. Cells were fixed with 3.7% formaldehyde and stained with crystal violet, and the numbers of cells on the bottom were counted. Data from three experiments done in triplicate are shown as mean ± standard error).

**Time-lapse video** **and migratory velocity.** The assessment of migratory velocity and migratory distance was conducted using time-lapse video images that were collected and stored as images using Metamorph, version 3.5, software ^8^. For time-lapse observation of cell movement, cells on 12-well plates were maintained in DMEM with 10% fetal calf serum (FCS) and HEPES. Cells were placed in a temperature and CO_2_ controlled incubator to maintain the temperature at 37°C and CO_2_ at 5%. The cell movement videos were taken at 5-min intervals by using a Nikon Eclipse TE-300 inverted microscope system. The cell movement velocity was determined by tracing the single cells at different time points using MetaMorph software.

**Cell Cycle Analysis.** Cell cycle parameters were determined using laser scanning cytometry. Cells were processed by standard methods using propidium iodide staining of cellular DNA. Each sample was analyzed by flow cytometry with a FACScan Flow Cytometer (Becton-Dickinson Biosciences, Mansfield, MA) using a 488 nm laser. Histograms were analyzed for cell cycle compartments using ModFit version 2.0 (Verity Software House, Topsham, ME). A minimum of 20,000 events were collected to maximize statistical validity of the compartmental analysis.

**Colony forming assays.** 4 x 10^3^ cells were plated in triplicate in 3 ml of 0.3% agarose (sea plaque) in complete growth medium overlaid on a 0.5% agarose base, also in complete growth medium. 2 weeks after incubation, colonies more than 50 µm in diameter were counted using an Omnicon 3600 image analysis system. The colonies were visualized after staining with 0.04% crystal violet in methanol for 1 to 2 h.

**Fluorescence resonance energy transfer (FRET**) **imaging.** HEK293T cells, co-transfected with 3xFLAG vector, cyclin D1 wild-type or cyclin D1^KE^ mutant and FRET reporters (pRaichu-RhoA, pRaichu-Cdc42 or pRaichu-Rac1 ^7, 17^), were cultured in a four-well chamber and imaged using a Zeiss laser-scanning microscope, LSM510META, with a 40× oil immersion Doc Plan-Neofluar lens objective (numerical aperture of 1.3). To detect FRET between CFP and YFP, we used time-lapse and lambda stack acquisition linked with the photobleaching command ^4^. In the time series, the cells were excited with the 454-nm laser at 7.5% power in order to limit photobleaching and images were recorded in 16 channels from 464 to 566 nm. Spectral images were recorded three times before photobleaching, the selected regions were illuminated with 100% 514-nm laser power of 100 times to photobleach YFP. Images were then recorded three more times with excitation by the 454-nm laser line. The fluorescence intensity in the regions of interest in the time-lapse experiments was measured with the LSM510META software. The FRET efficiency was calculated as FRET% = (Fa-Fb)/Fb x 100%. Fa was the fluorescence intensity at 481 nm after photobleaching and Fb was before photobleaching. The FRET image was shown as a ratio image of 481 nm/534 nm which was represented as the CFP/YFP signals.

**Western blot**. Western blot analyses were conducted ^8^ using antibodies to cyclin D1 (sc-20044, Santa Cruz), β-actin (sc-47778, Santa Cruz), Akt1 (sc-5298, Santa Cruz), pAkt1/2/3 Ser473 (sc-7895-R, Santa Cruz), pAkt1 Ser473 (#9018, CST), Na^+^/K^+^-ATPase (D4Y7E, #23565, Cell Signaling), Histone H2A (#9718S, Cell Signaling). Mouse anti-FLAG (M2), mouse anti-vinculin (hVIN-1) antibodies were from Sigma (St. Louis, MO).

**Immunostaining.** Immunofluorescence staining and confocal microscopy of cultured cells was conducted as described previously ^5^. Chromogen immunostaining of human breast cancer samples was conducted on the breast tissue with the Ventana Benchmark autostainer using deidentified archival tissue which are exempt from review by the Thomas Jefferson University Institutional Review Board. The slides of the tumor tissue were scanned with the Aperio digital analysis system. The subcellular distribution was assigned using the standard Aperio digital analysis algorithm for cell-membrane staining. Inflammatory breast cancers were classified based on the patient’s clinical appearance as determined by the primary oncologist. The cells in four-well chamber slides were fixed with 4% paraformaldehyde for 10 min at room temperature (RT) and cold methanol for 5 min at -20°C. The slides were then treated with 0.2% Triton X-100 for 5 min at RT and blocked with 2% BSA overnight at 4^o^C. The primary antibodies used were cyclin D1 (sc-20044, Santa Cruz),), PACSIN II (sc-5574, Santa Cruz), tyrosine phosphorylated PACSIN II (44-722G, Life Tech), F-actin (ab205, Abcam). The secondary antibodies used were Alexa Fluor 488-conjugated F(ab')2 fragment of goat anti-rabbit immunoglobulin G (IgG) (Molecular Probes, Inc.) (1/500), Rhodamine-conjugated F(ab')2 fragment of goat anti-rabbit immunoglobulin G (IgG) (Jackson ImmunoResearch Laboratories, Inc) (1/500). The samples were visualized on a Zeiss LSM 510 META Confocal Microscope with a 63x objective.

Fluorescence-based immunohistochemistry for cyclin D1 multiplexed with pan-cytokeratin and DAPI counterstain was performed as previously described ^6, 10, 11^ on a tissue microarray containing cores of 50 de-identified ERα-positive breast cancer specimens provided by the Medical College of Wisconsin Tissue Bank under an IRB-approved protocol.  Cyclin D1 was detected in clinical breast cancer specimens by immunofluorescence-immunohistochemistry (IF-IHC) performed on an Omnis autostainer (Dako/Agilent) with rabbit monoclonal cyclin D1 antibody (Dako, M3635), diluted 1:200 and coincubated with the mouse monoclonal anti-pan-cytokeratin (clone AE1/AE3, DAKO, 1:100) for 20 minutes. Prior antigen retrieval was conducted using the built-in Omnis retrieval module with citric acid buffer (pH 6.0). High-resolution images for three-color presentation were scanned on the Pannoramic Flash 250 (3DHistech) ^6, 10, 11^.

**Live cell Akt activity monitoring** ^14^**.** *Cyclin D1* *wt* and *cyclin D1^-/-^* MEFs were maintained at 37^o^C and 5% CO_2_ in DMEM medium supplemented with 10% fetal bovine serum, 100 unit/mL penicillin, and 100 mg/mL streptomycin. The cells (10^5^ cell/well) were cultured in glass bottom dishes overnight and transfected with 5 mM of each LS456, using GeneJuice transfection reagent (Novagen,Madison, WI) in the dish for 18 h at 37^o^C. The transfected cells were treated with 150 nM insulin in Tris-buffered saline and imaged at 30^o^C using an FV1000 confocal microscope with UPLanApo/IR 60X/1.20W objective lens (Olympus, Center Valley, PA).  The treated cells were incubated with 150 nM of insulin. Imaging was conducted at different time points. The mean fluorescence intensity (n58) (Ex/Em=633/670-730 nm; Ex/Em=785/805–830 nm) in the dishes was determined with FV1000 software. All of the fluorescence intensity changes at each time point were normalized to the positive control (0 min). The image of LS542 (**Figure S7**) were acquired with LI-COR Pearl Imager (Lincoln, NE) at Ex/Em of 685/705 nm and 785/810 nm channels. Live cell imaging studies were conducted at 30^o^C, which is the default room temperature for the imaging platform of the microscope. All images within the same time series were recorded from the same area of the slides. However, as the live cells moved during the imaging sessions, eight representative cells were used to quantify the fluorescence using Olympus FV1000 software.

REFERENCES

1 Albanese C, D'Amico M, Reutens AT, Fu M, Watanabe G, Lee RJ *et al* (1999). Activation of the *cyclin D1* gene by the E1A-associated protein p300 through AP-1 inhibits cellular apoptosis. *J Biol Chem* **274:** 34186-34195.

2 Casimiro MC, Crosariol M, Loro E, Ertel A, Yu Z, Dampier W *et al* (2012). ChIP sequencing of cyclin D1 reveals a transcriptional role in chromosomal instability in mice. *J Clin Invest* **122:** 833-843.

3 Casimiro MC, Di Sante G, Ju X, Li Z, Chen K, Crosariol M *et al* (2016). Cyclin D1 Promotes Androgen-Dependent DNA Damage Repair in Prostate Cancer Cells. *Cancer Res* **76:** 329-338.

4 Jiao X, Zhang N, Xu X, Oppenheim JJ, Jin T (2005). Ligand-induced partitioning of human CXCR1 chemokine receptors with lipid raft microenvironments facilitates G-protein-dependent signaling. *Mol Cell Biol* **25:** 5752-5762.

5 Jiao X, Katiyar S, Liu M, Mueller SC, Lisanti MP, Li A *et al* (2008). Disruption of c-Jun reduces cellular migration and invasion through inhibition of c-Src and hyperactivation of ROCK II kinase. *Mol Biol Cell* **19:** 1378-1390.

6 Jiao X, Velasco-Velazquez MA, Wang M, Li Z, Rui H, Peck AR *et al* (2018). CCR5 Governs DNA Damage Repair and Breast Cancer Stem Cell Expansion. *Cancer Res* **78:** 1657-1671.

7 Lam AJ, St-Pierre F, Gong Y, Marshall JD, Cranfill PJ, Baird MA *et al* (2012). Improving FRET dynamic range with bright green and red fluorescent proteins. *Nat Methods* **9:** 1005-1012.

8 Li Z, Wang C, Jiao X, Lu Y, Fu M, Quong AA *et al* (2006). Cyclin D1 regulates cellular migration through the inhibition of thrombospondin 1 and ROCK signaling. *Mol Cell Biol* **26:** 4240-4256.

9 Li Z, Jiao X, Wang C, Shirley LA, Elsaleh H, Dahl O *et al* (2010). Alternative cyclin D1 splice forms differentially regulate the DNA damage response. *Cancer Res* **70:** 8802-8811.

10 Peck AR, Girondo MA, Liu C, Kovatich AJ, Hooke JA, Shriver CD *et al* (2016). Validation of tumor protein marker quantification by two independent automated immunofluorescence image analysis platforms. *Mod Pathol* **29:** 1143-1154.

11 Pestell TG, Jiao X, Kumar M, Peck AR, Prisco M, Deng S *et al* (2017). Stromal cyclin D1 promotes heterotypic immune signaling and breast cancer growth. *Oncotarget* **8:** 81754-81775.

12 Rao M, Casimiro MC, Lisanti MP, D'Amico M, Wang C, Shirley LA *et al* (2008). Inhibition of cyclin D1 gene transcription by Brg-1. *Cell Cycle* **7:** 647-655.

13 Razandi M, Alton G, Pedram A, Ghonshani S, Webb P, Levin ER (2003). Identification of a structural determinant necessary for the localization and function of estrogen receptor alpha at the plasma membrane. *Mol Cell Biol* **23:** 1633-1646.

14 Shen D, Bai M, Tang R, Xu B, Ju X, Pestell RG *et al* (2013). Dual fluorescent molecular substrates selectively report the activation, sustainability and reversibility of cellular PKB/Akt activity. *Sci Rep* **3:** 1697.

15 Soutoglou E, Misteli T (2008). Activation of the cellular DNA damage response in the absence of DNA lesions. *Science* **320:** 1507-1510.

16 Wang C, Pattabiraman N, Zhou JN, Fu M, Sakamaki T, Albanese C *et al* (2003). Cyclin D1 repression of peroxisome proliferator-activated receptor gamma expression and transactivation. *Mol Cell Biol* **23:** 6159-6173.

17 Yoshizaki H, Ohba Y, Kurokawa K, Itoh RE, Nakamura T, Mochizuki N *et al* (2003). Activity of Rho-family GTPases during cell division as visualized with FRET-based probes. *J Cell Biol* **162:** 223-232.

**Supplemental Figure legends**

**Figure S1. Cyclin D1 is located in the cytoplasmic membrane.** (A,B). The human diploid fibroblast cell line (MRC-5) was stained for cyclin D1, PACSIN II, and F-actin. Merged images demonstrate the presence of cyclin D1 with PACSIN II and F-actin. The size bar is 5 μm.

**Figure S2. Cyclin D1 is located in the cytoplasmic membrane** (A,B). Cyclin D1 co-staining with tyrosine phosphorylated Paxillin (Y118) and F-actin in MRC-5 cells. Focal contacts are identified by the tyrosine phosphorylated Paxillin.

**Figure S3. Membrane-associated cyclin D1 in human breast cancer.** (A). The representative image for cyclin D1 IHC staining conducted of inflammatory breast cancer (IBC) and non-inflammatory breast cancer patient samples. Membrane-associated cyclin D1 is highlighted (white arrows) in a representative example of IBC. (B). The percent of patients with membrane localized cyclin D1 in breast cancer patients. 5 of 6 IBC patients and 2 of 17 non-IBC patients show membrane-associated cyclin D1 (P<0.005 by Z-test). (C-F). Examples of membrane-localized cyclin D1 in histological sections of non-IBC breast cancer specimens assessed further by immunofluorescence. Four cases of estrogen receptor α-positive breast cancer immunostained for cyclin D1 (red color) and pan-cytokeratin (carcinoma cell marker, green color underexposed to accentuate cell perimeters/boundaries). Cell nuclei were visualized with DAPI (blue color). Cells in which cyclin D1 is present in the cell membrane are indicated by arrows and are predominantly yellow due to merging of red and green signals. In contrast, nuclear cyclin D1 is predominantly pink due to merging of red and blue. Scale bars = 20 µm.

**Figure S4. Membrane-associated cyclin D1 promotes cellular migratory velocity.** Transwell migration assays of serum starved MCF-7 cells transfected with expression vectors encoding either cyclin D1^WT^, nuclear-localized cyclin D1^NUC^ (cherry-CD1^NUC^) or (membrane-associated cyclin D1^MEM^ (PECFP-CD1^MEM^). (A) Data (N=2) for each condition, representative of N=16 are shown as transwell migration (cell per field) or (B) fold change in migrated cells compared with control vector.

**Figure S5. Immunofluorescence of membrane- and nuclear-localized cyclin D1.** (A). Confocal and (B). Z series confocal microscopy images of HEK293T cells transfected with pECFP-D1^MEM^ and (C,D) cherry-D1^NUC^. The representative images of pECFP-D1^MEM^ and cherry-D1^NUC^ transfected HEK293T cells shows the predominantly membrane localized cyclin D1-ECFP and (C,D) predominantly nuclear located cyclin D1 cherry red fluorescent fusion protein.

**Fig. S6. Membrane targeted cyclin D1 induces the immediate early gene c-*Fos*-LUC and *cyclin D1*-LUC.** HEK293T cells were transfected with either pECFP-D1^MEM^ or cherry-D1^NUC^ and the luciferase reporter genes as shown. The induction of luciferase activity is shown relative to equal molar amounts of the control expression vector and compared with wildtype cyclin D1. Data are shown as mean ± SEM.

**Fig. S7.** **Selective phosphorylation of LS456 by insulin activation requires cyclin D1**. (A). The fluorescence intensity of LS456 in either *cyclin D1^+/+^* MEFs or *cyclin D1^-/-^* MEFs (below) is decreased in the 800 nm channel and increased in the 700 nm channel, with delayed and reduced induction changes in *cyclin D1^-/-^* cells. (B-D). Quantitative analysis of the FI channel in wild type and *cyclin D1* deficient cells. Fluorescent images in cells were superimposed on differential interference contrast images with all figures shown at the same scale bar (20 μm). All images within the same time series were recorded from the same area of the slides. However, as the live cells moved during the imaging sessions, eight representative cells were used to quantify the fluorescence using Olympus FV1000 software. The data is shown as mean ± standard error for n=8 separate cells.
