# Supplementary material for: The membrane-associated form of cyclin D1 enhances cellular invasion
Source: Oncogenesis. 2020 Sep 18;9(9):83. doi: 10.1038/s41389-020-00266-y (PMC7501870; doi:10.1038/s41389-020-00266-y)
Supplement: Supplementary file 2 — Supplemental Figures [file 41389_2020_266_MOESM2_ESM.pptx]

## Slide 1
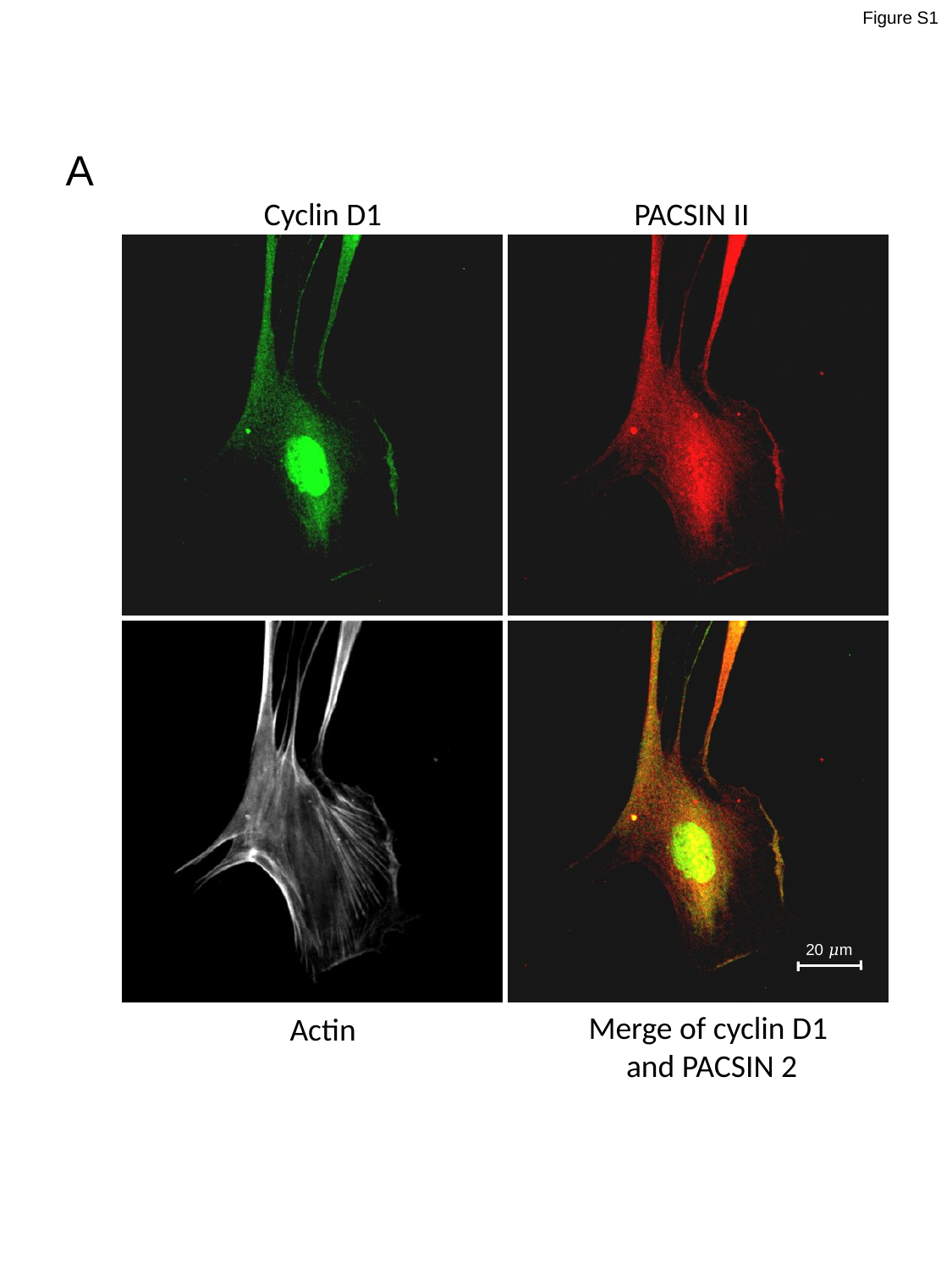

Figure S1
A
Cyclin D1
PACSIN II
20 𝜇m
Merge of cyclin D1
 and PACSIN 2
Actin

## Slide 2
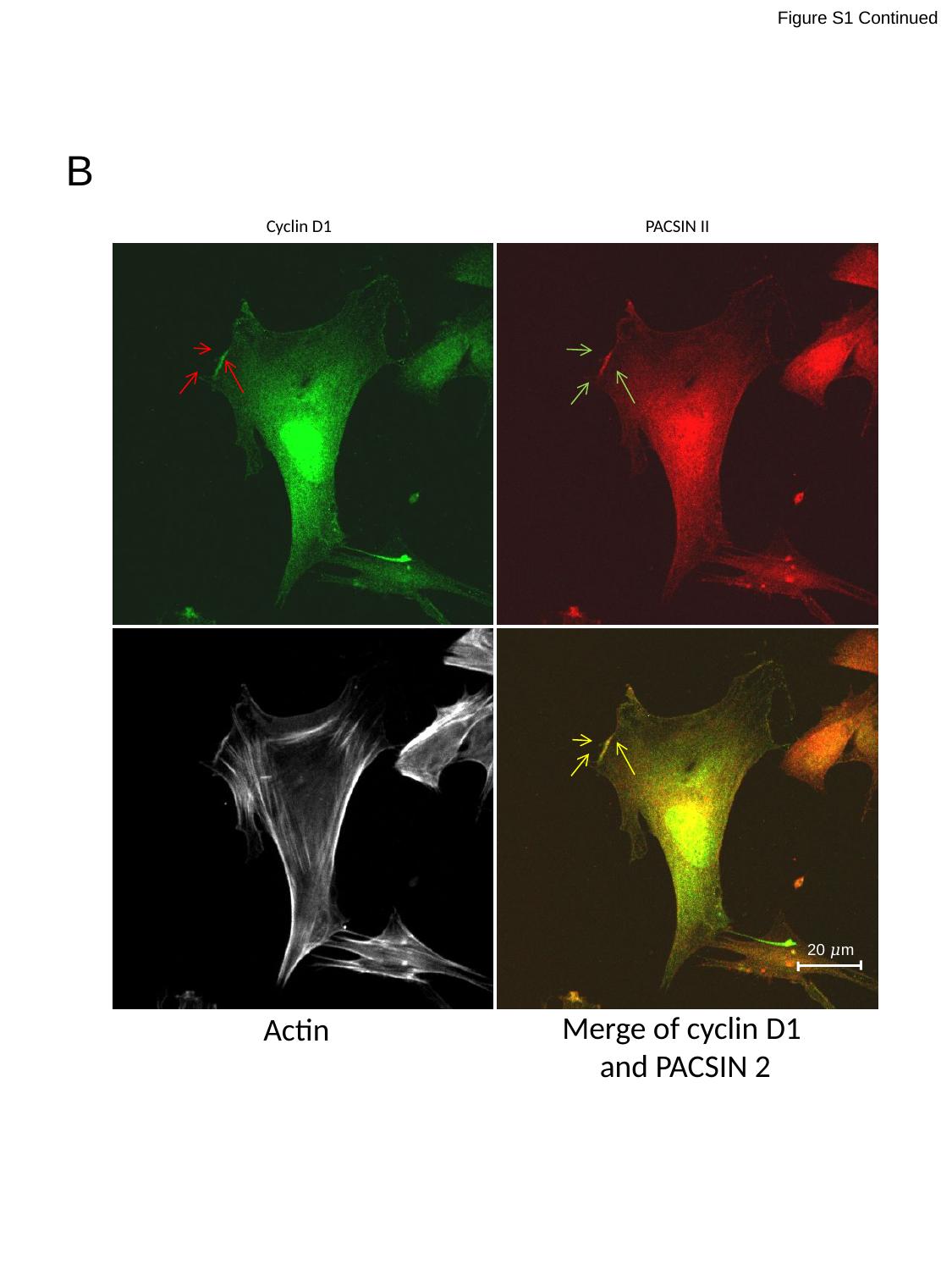

Figure S1 Continued
B
Cyclin D1
PACSIN II
20 𝜇m
Merge of cyclin D1
 and PACSIN 2
Actin

## Slide 3
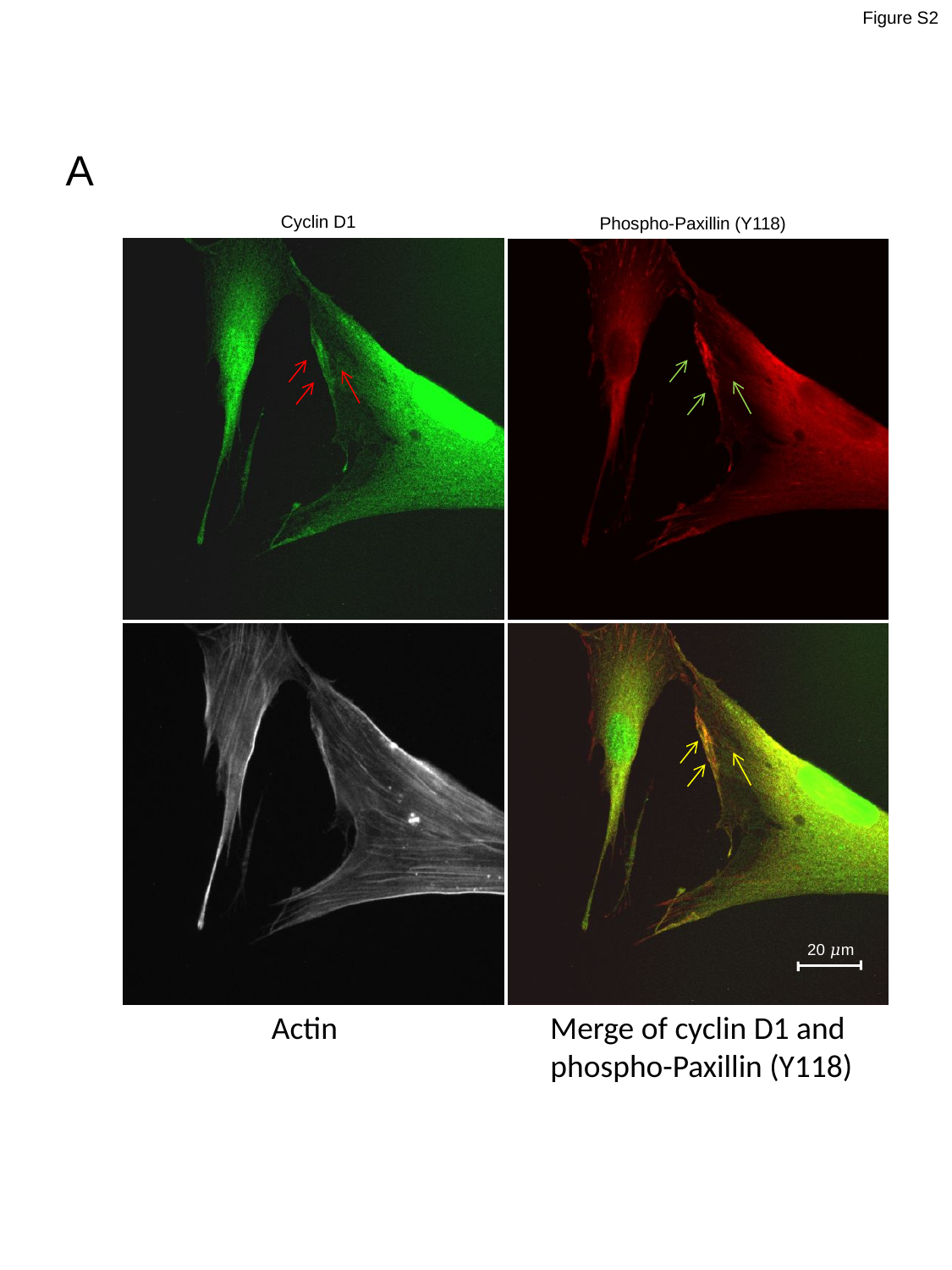

Figure S2
A
Cyclin D1
Phospho-Paxillin (Y118)
20 𝜇m
Actin
Merge of cyclin D1 and
phospho-Paxillin (Y118)

## Slide 4
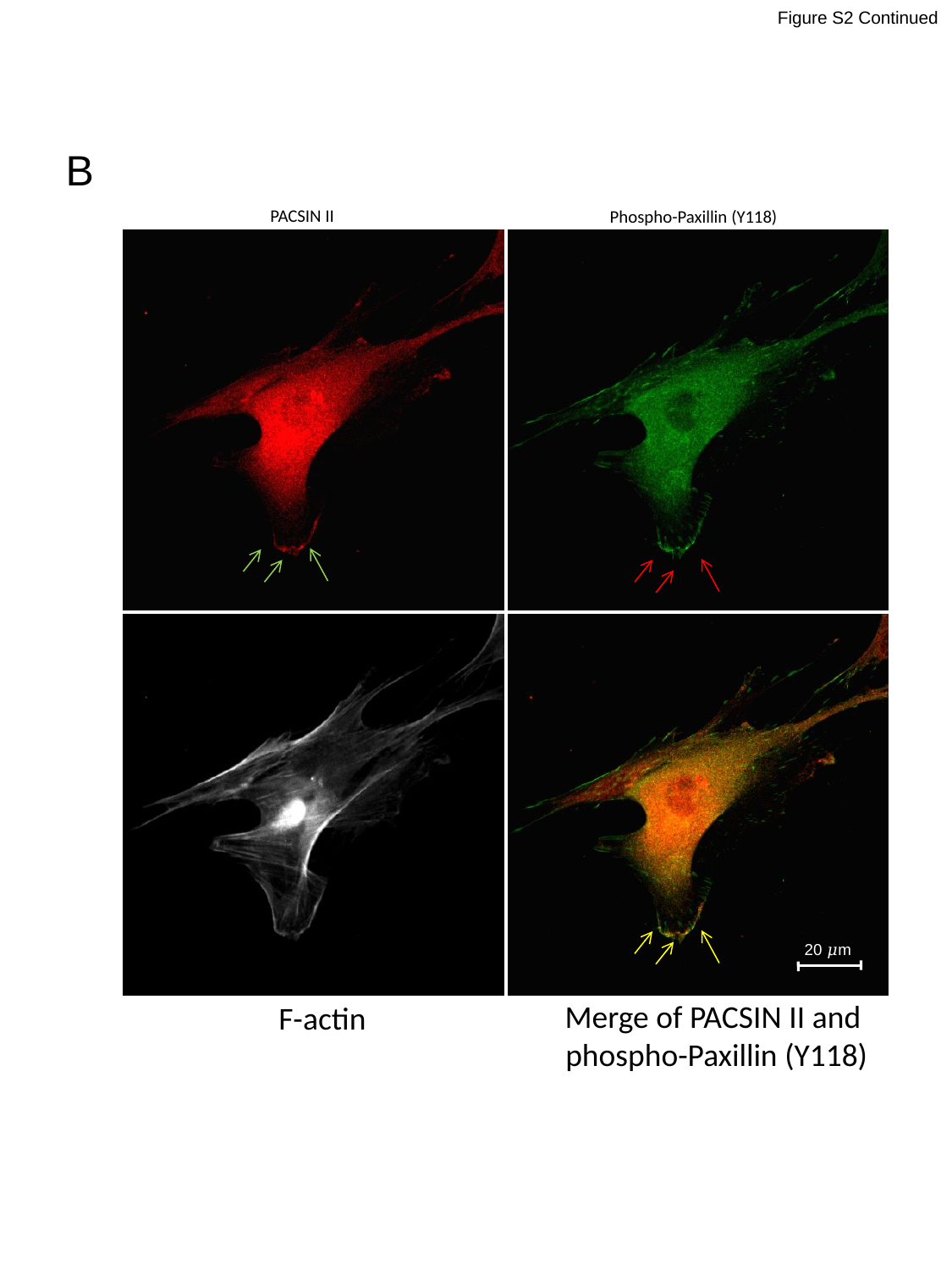

Figure S2 Continued
B
PACSIN II
Phospho-Paxillin (Y118)
20 𝜇m
Merge of PACSIN II and
phospho-Paxillin (Y118)
F-actin

## Slide 5
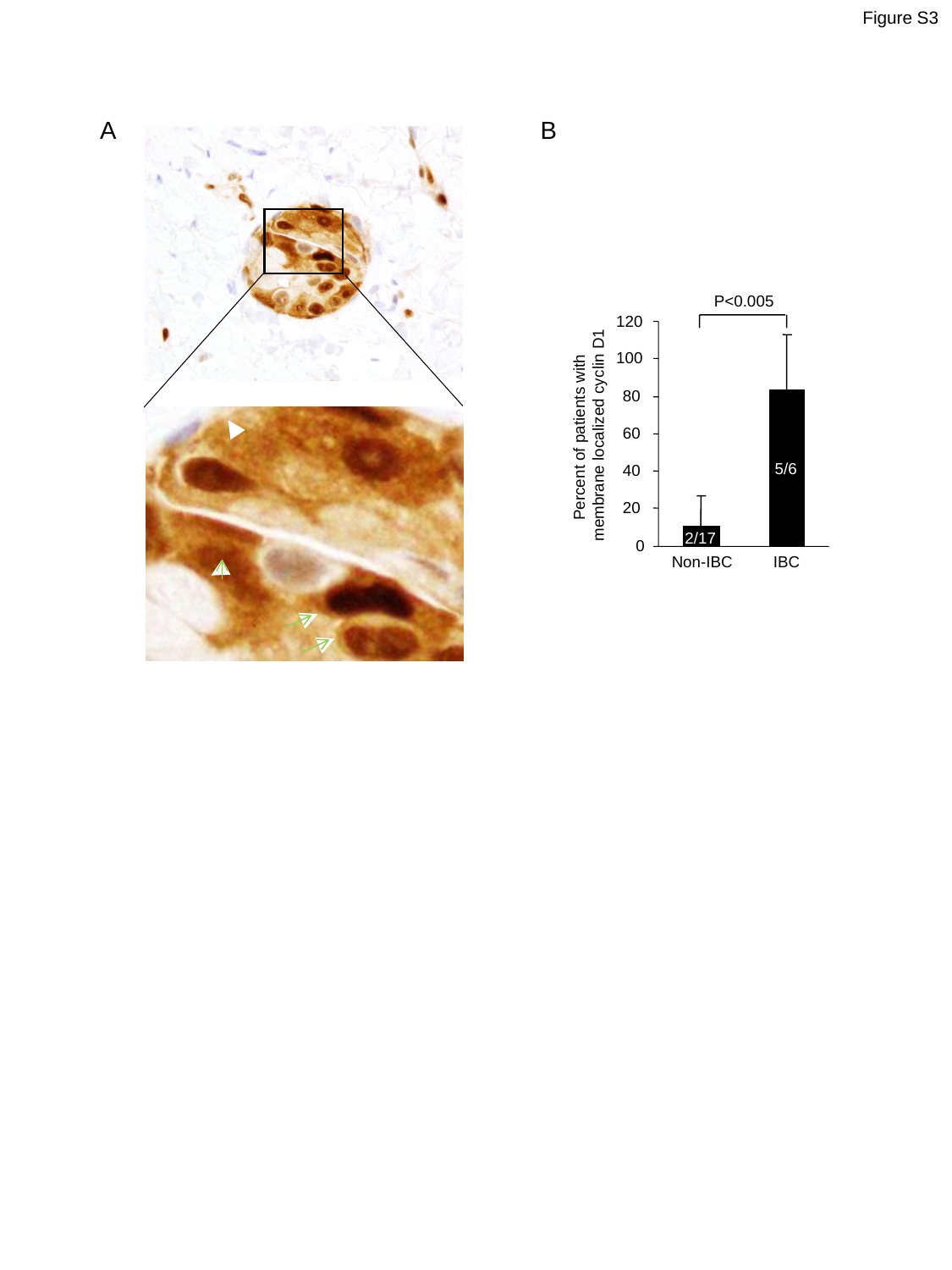

Figure S3
A
B
P<0.005
120
100
80
Percent of patients with
membrane localized cyclin D1
60
5/6
40
20
2/17
0
Non-IBC
IBC

## Slide 6
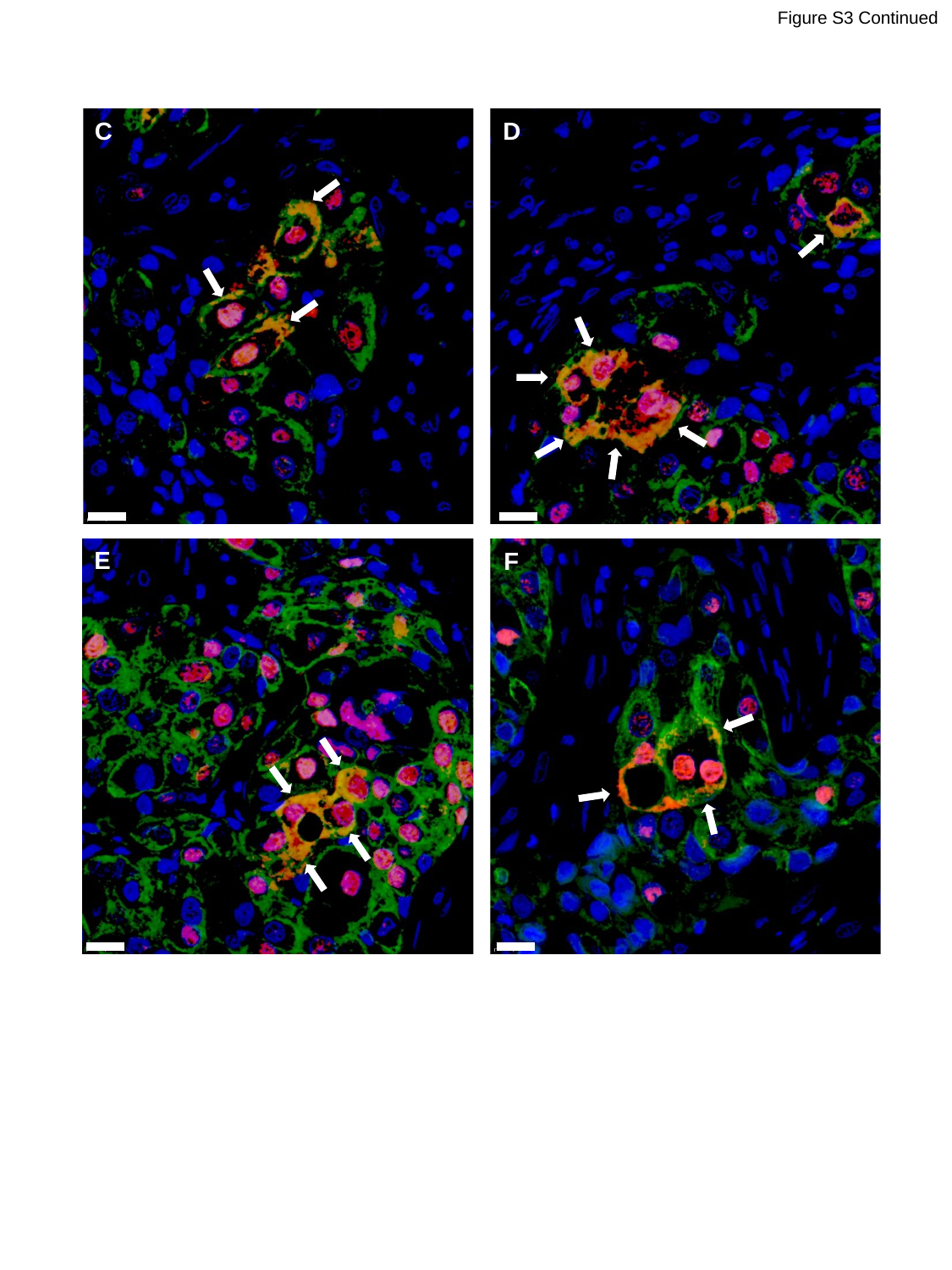

Figure S3 Continued
C
D
E
F

## Slide 7
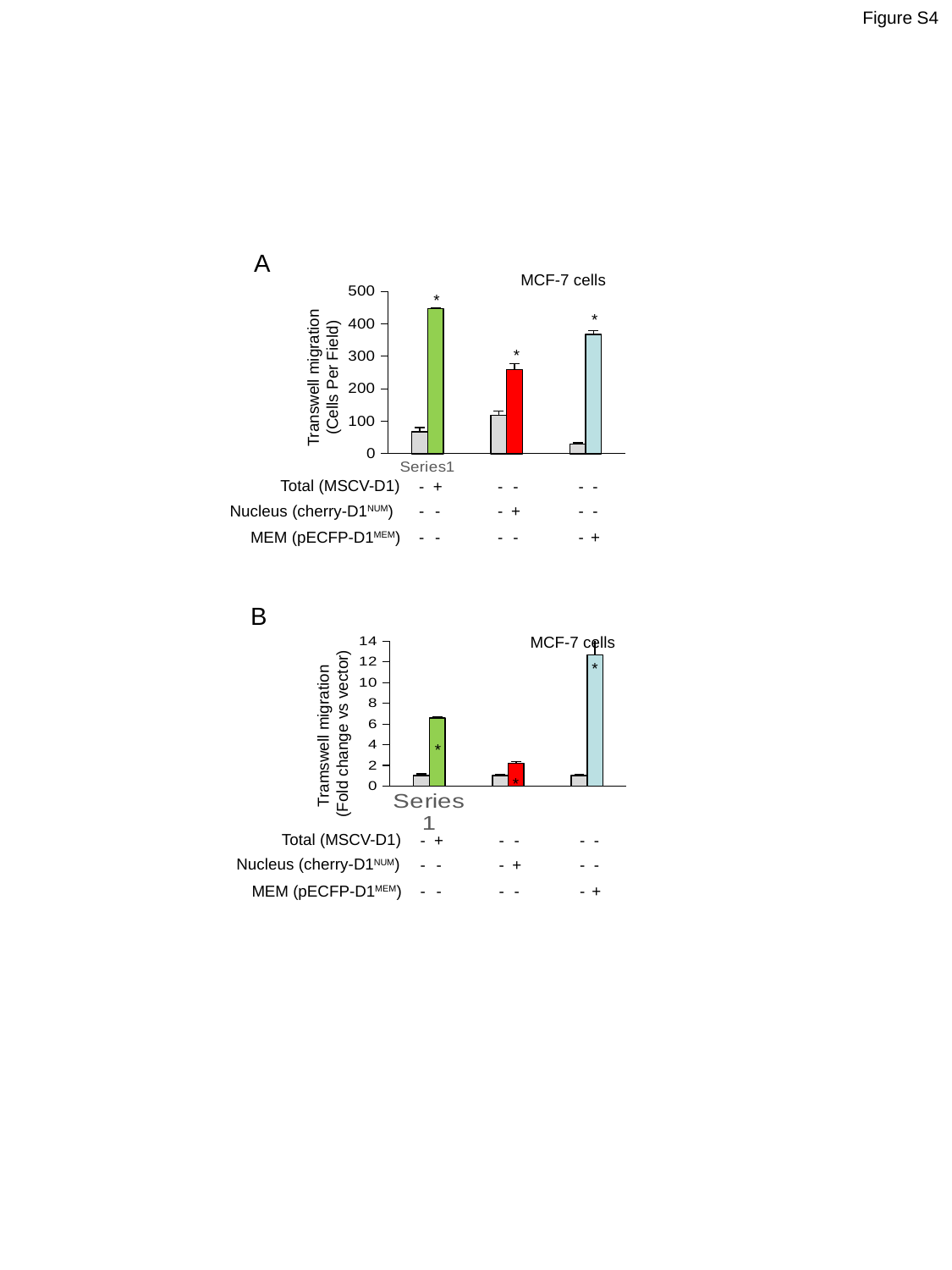

Figure S4
A
MCF-7 cells
### Chart
| Category | vector | Di |
|---|---|---|
| | 67.5 | 446.0 |
| | 118.0 | 258.5 |
| | 29.0 | 367.5 |*
*
*
Transwell migration
(Cells Per Field)
Total (MSCV-D1)
-
+
-
-
-
-
Nucleus (cherry-D1NUM)
-
-
-
+
-
-
MEM (pECFP-D1MEM)
-
-
-
-
-
+
B
MCF-7 cells
### Chart
| Category | | |
|---|---|---|
| | 1.0 | 6.607407407407393 |
| | 1.0 | 2.190677966101695 |
| | 1.0 | 12.67241379310345 |*
Tramswell migration
(Fold change vs vector)
*
*
Total (MSCV-D1)
-
+
-
-
-
-
Nucleus (cherry-D1NUM)
-
-
-
+
-
-
MEM (pECFP-D1MEM)
-
-
-
-
-
+

## Slide 8
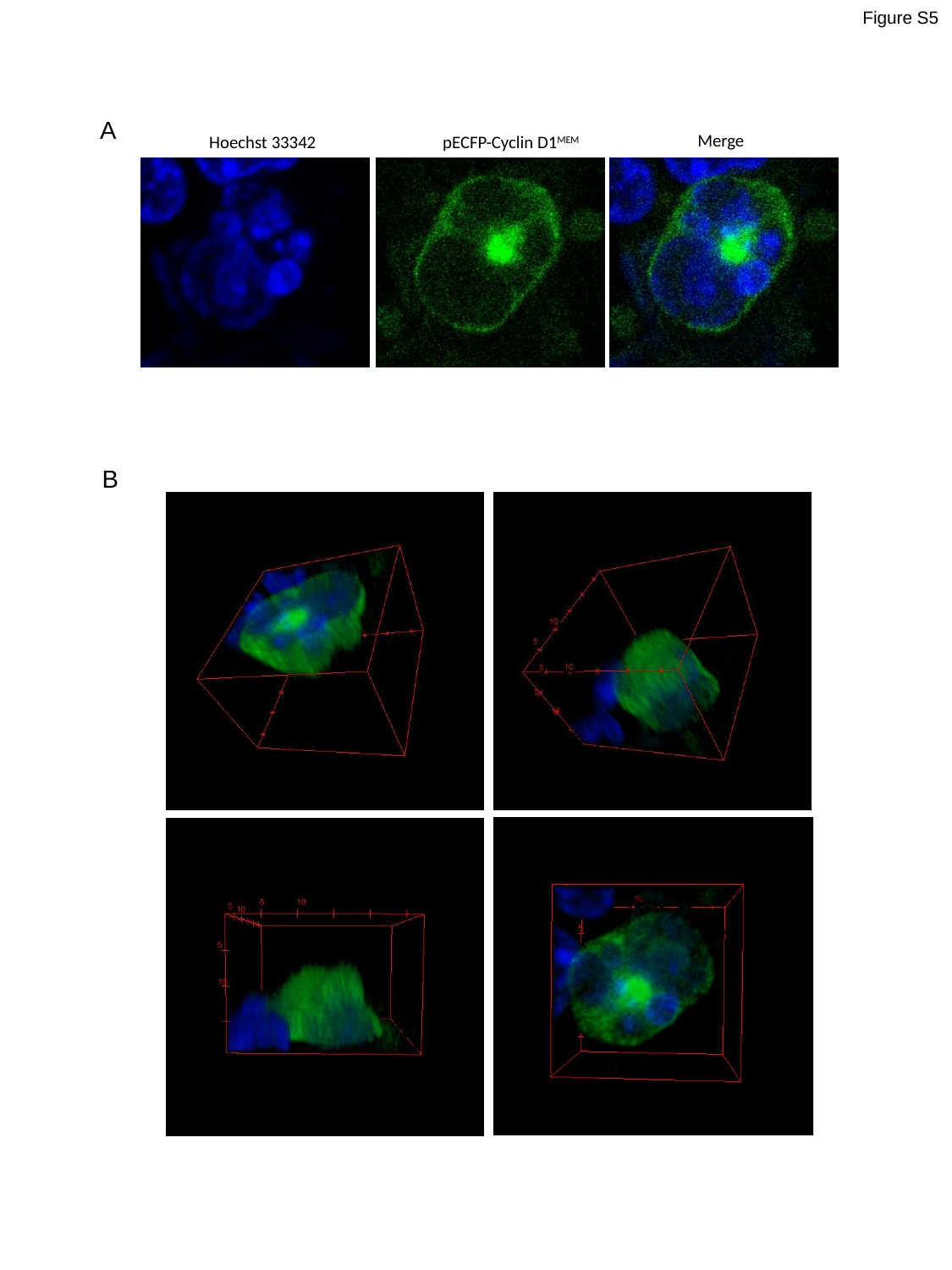

Figure S5
A
Merge
Hoechst 33342
pECFP-Cyclin D1MEM
Nucleus (cherry-D1NUC)
B

## Slide 9
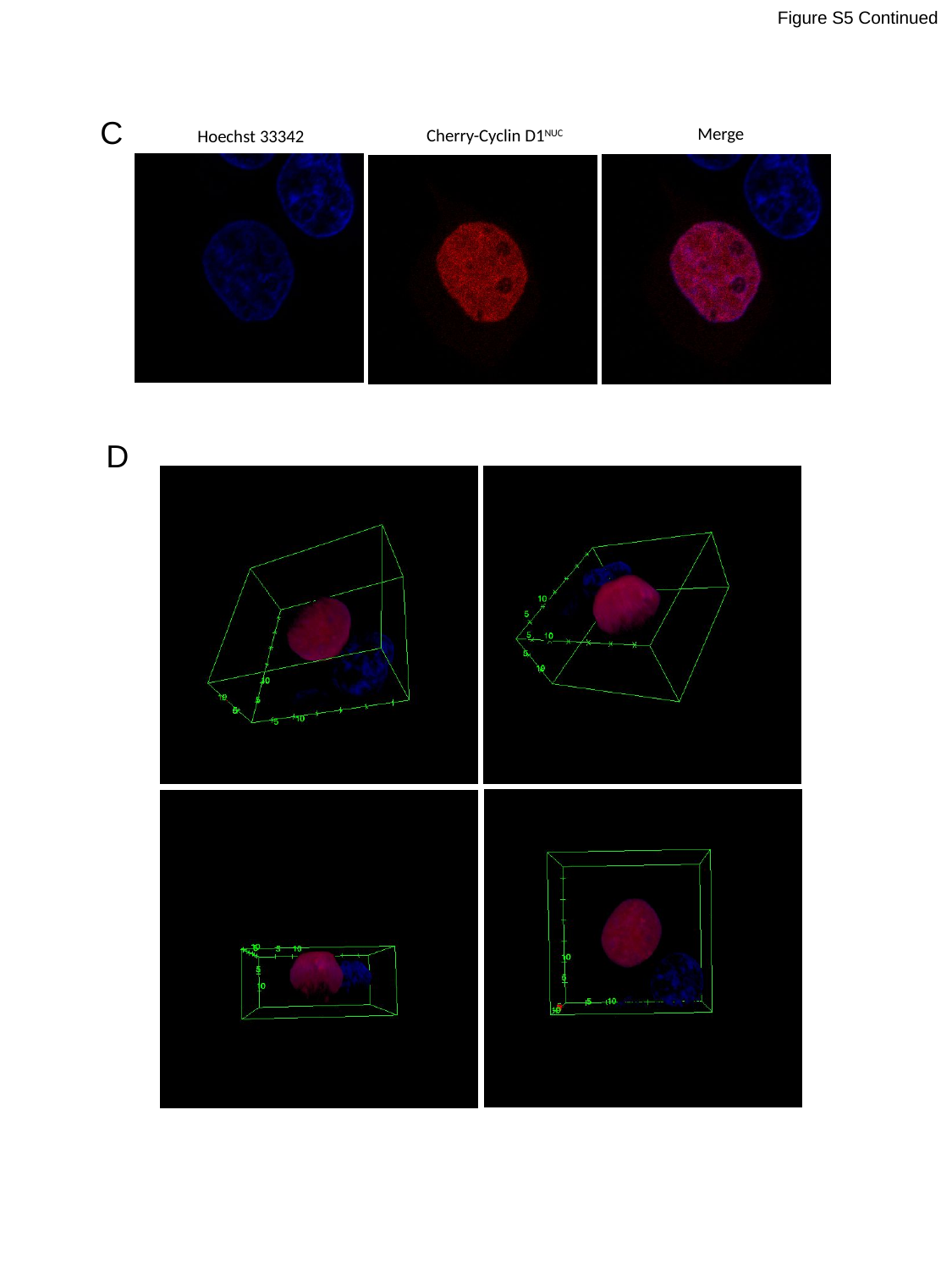

Figure S5 Continued
C
Merge
Cherry-Cyclin D1NUC
Hoechst 33342
D

## Slide 10
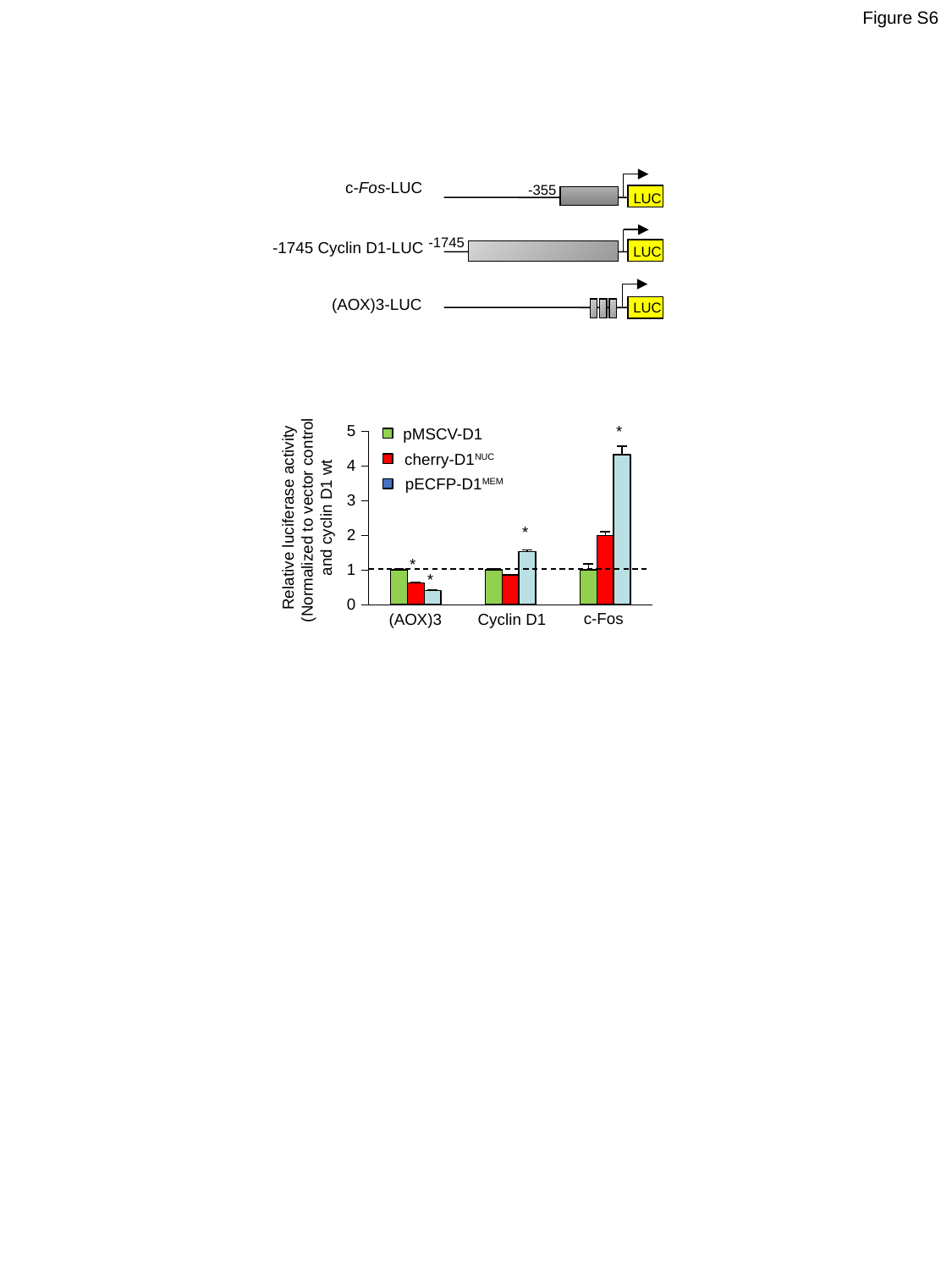

Figure S6
c-Fos-LUC
-355
LUC
-1745
-1745 Cyclin D1-LUC
LUC
(AOX)3-LUC
LUC
*
*
*
*
pMSCV-D1
### Chart
| Category | Wt-CycD1 | nuc-CycD1 | mem-CycD1 |
|---|---|---|---|
| AOX | 1.0 | 0.63119879890228 | 0.411601349975816 |
| Cyclin D1 | 1.0 | 0.851811091856114 | 1.524710636216514 |
| c-Fos | 1.0 | 1.993402763416322 | 4.31983435634471 |
cherry-D1NUC
pECFP-D1MEM
Relative luciferase activity
(Normalized to vector control
and cyclin D1 wt
c-Fos
(AOX)3
Cyclin D1

## Slide 11
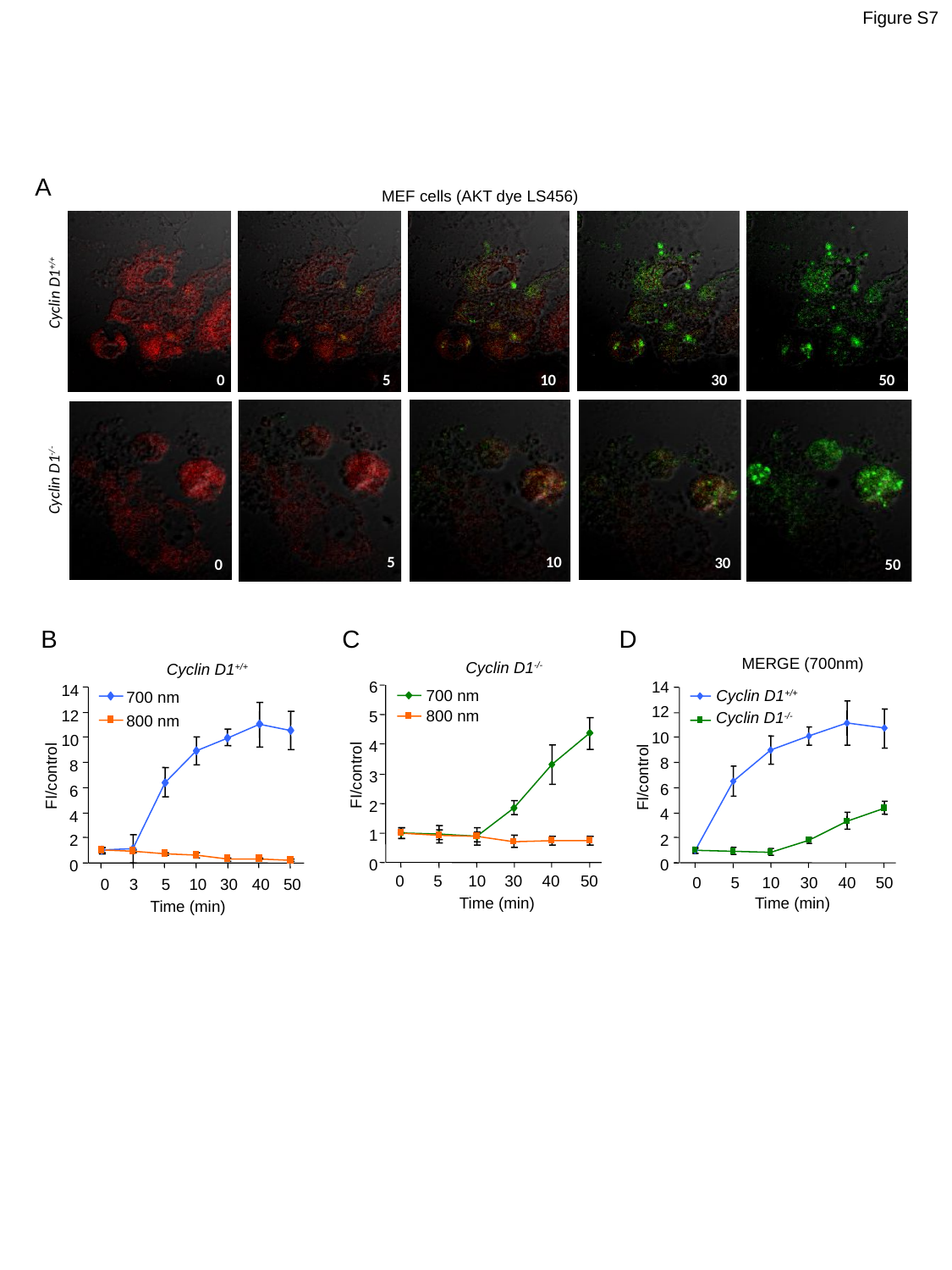

Figure S7
A
MEF cells (AKT dye LS456)
Cyclin D1+/+
0
5
10
30
50
Cyclin D1-/-
5
10
30
50
0
0
B
C
D
MERGE (700nm)
Cyclin D1-/-
Cyclin D1+/+
14
6
Cyclin D1+/+
14
700 nm
700 nm
800 nm
Cyclin D1-/-
12
12
800 nm
5
10
10
4
8
8
FI/control
FI/control
FI/control
3
6
6
2
4
4
1
2
2
0
0
0
0
5
10
30
40
50
0
5
10
30
40
50
0
3
5
10
30
40
50
Time (min)
Time (min)
Time (min)
